# Effects of multicomponent exercise injury prevention programs on adolescent team athletes (10–19 years old): a systematic review and meta-analysis

**DOI:** 10.3389/fped.2025.1561993

**Published:** 2026-01-07

**Authors:** Honghao Liu, Xiaoming Liu, Long Yin

**Affiliations:** College of Physical Education, Hunan Normal University, Changsha, China

**Keywords:** multicomponent exercise, injury prevention program, adolescent, team athletes, meta-analysis

## Abstract

**Objective:**

The aim of this study was to assess the efficacy of multicomponent exercise injury prevention programs in adolescent team athletes and to determine the optimal training programs characteristics, including program duration and potential sex-related differences, through subgroup analysis.

**Methods:**

A thorough literature search was conducted in PubMed, Web of Science, Embase, EBSCOhost, and the Cochrane Library, covering all publications from their inception to 20 July 2024.

**Results:**

This meta-analysis of 16 randomized controlled trials evaluated the effectiveness of multicomponent exercise interventions in reducing sports-related injuries among adolescent team athletes and identified optimal training components, duration, and potential sex-related differences. The results revealed that these programs significantly reduced injuries across various body regions: total injuries by 35% [incidence rate ratio (IRR) = 0.65, 95% confidence interval (CI): 0.54–0.77], lower-extremity injuries by 33% (IRR = 0.67, 95% CI: 0.57–0.80), knee injuries by 22% (IRR = 0.78, 95% CI: 0.66–0.92), ankle injuries by 38% (IRR = 0.62, 95% CI: 0.47–0.81), and upper-extremity injuries by 32% (IRR = 0.68, 95% CI: 0.40–1.17). These programs also reduced acute injuries by 32% (IRR = 0.68, 95% CI: 0.57–0.81) and overuse injuries by 39% (IRR = 0.61, 95% CI: 0.49–0.76). Findings suggest that interventions under 20 min (IRR = 0.59, 95% CI: 0.44–0.79) and incorporating warm-up, jumping/plyometric, strength, agility, and balance are most effective (IRR = 0.55, 95% CI: 0.41–0.73). Subgroup analyses revealed stronger effects in females (IRR = 0.56, 95% CI: 0.35–0.88) than in males (IRR = 0.66, 95% CI: 0.55–0.80) and found greater effectiveness in interventions lasting under 20 min compared to those lasting 20 min or more (IRR = 0.70, 95% CI: 0.57–0.86). Notably, most research training programs include balance and strength training.

**Conclusion:**

Multicomponent sports intervention programs are effective in reducing injury incidence among adolescent team athletes. Subgroup analyses identified significant sex-related differences and confirmed the optimal training duration (<20 min) as well as the most effective training combination (warm-up, jumping/plyometric, strength, agility, and balance training). Strength training (focusing on the hamstrings and core) and balance training are recommended for inclusion in a multicomponent exercise injury prevention program. However, the low quality of current evidence underscores the need for rigorous studies to confirm these findings.

**Systematic Review Registration:**

CRD42024580565.

## 1 Introduction

Injury prevention remains a central focus of research in the field of sports (1). To safeguard the health of athletes and enhance their performance, researchers have actively pursued various strategies aimed at reducing injury risks in sports. As competition intensity and duration continue to increase, the likelihood of injury among athletes also increases, underscoring the critical importance of injury prevention research (2–5).

Studies have demonstrated that adolescents experience a high incidence of exercise-related injuries (6–8). Several factors contribute to this phenomenon. Adolescence is a critical stage of physical, sociopsychological, and cognitive development, as well as a period of physical and psychological vulnerability. This vulnerability is primarily caused by three factors: the characteristics of the musculoskeletal system, the influence of adolescent hormones, and the lack of coordination between physical and cognitive development. These factors increase the risk of injury when adolescents engage in sports. Although the physical and intellectual development of adolescents progresses rapidly, these developmental levels are not yet fully aligned with the demands of high-intensity competitive sports, thereby increasing the risk of injury (9, 10). Moreover, adolescents may struggle to prevent sport-specific injuries due to differences in how they acquire and apply sports skills. For example, improper landings in basketball can cause injury. Inadequate attention to recovery from sports injuries by parents and coaches can further exacerbate the risk of injury (11, 12).

Previous studies have examined injury prevention training modalities—including Single-Mode Training, FIFA11+, and Neuromuscular Training—in adolescents (13–18). Both FIFA11+ and Neuromuscular Training are classified as multicomponent injury prevention programs, which typically combine two or more training methods, such as warm-up, plyometrics, strength, agility, speed, stretching, and balance exercises (19). Previous meta-analyses have explored the effects of these programs in adolescents, but their focus differs from the present study. For example, Rössler et al. (20) included both single-intervention and multicomponent intervention programs in their study design, but did not conduct comparative analyses of multicomponent interventions. During subgroup analysis, the researchers of that study only compared the effects of “balance exercises” vs. “jumping or plyometric exercises” individually, without further analyzing combinations of different exercise types. Consequently, the optimal exercise intervention for this population remains unclear. The study included athletes “under 19 years of age,” but our meta-analysis specifically focused on adolescents as defined by the World Health Organization (10–19 years). This approach ensures greater homogeneity in our sample, allowing us to concentrate on the unique physiological and developmental characteristics of this age group. Similarly, the study by Soomro et al. (21) focused solely on overall injury prevention outcomes without examining effects on preventing injuries to specific body parts or analyzing intervention efficacy across different injury mechanisms. Their subgroup analysis compared the “FIFA11+” program with the “non-FIFA11+” program. The study did not compare the effectiveness of intervention programs with different combinations of exercise components, making it difficult to determine the optimal combination for preventing specific injuries.

To address these existing research gaps, the present study systematically evaluated the specific effects of multicomponent injury prevention programs on adolescent team sport athletes. Unlike previous reviews, our analysis focuses on determining how these interventions affect injuries to specific body parts and different injury mechanisms, including both acute injuries and overuse injuries. This study also used subgroup analysis for the first time to determine the optimal training combination, intervention duration, and sex-related differences. These findings may help adolescent athletes, their parents, and coaches better understand sports injuries and implement effective strategies to reduce their incidence.

## 2 Methods

### 2.1 Protocol and registration

This systematic review and meta-analysis was conducted in accordance with the updated guidelines of the Preferred Reporting Items for Systematic Reviews and Meta-Analyses (PRISMA) 2020 statement (22). The study has been registered with PROSPERO (CRD42024580565).

### 2.2 Eligibility criteria

In this study, the inclusion and exclusion of articles for the meta-analysis were determined according to the Population, Intervention, Comparison, Outcome, Study design (PICOS) framework. No restrictions were applied regarding the language or publication year of the included articles. The inclusion criteria were as follows: (a) Participants: adolescent team athletes (aged 10–19 years), with no restrictions on sex; (b) Intervention: studies involving two or more exercise interventions (19); (c) Control: the control group continued their usual training; (d) Outcome: injury incidence rates across different body regions and injury mechanisms (acute injuries and overuse injuries); and (e) Study type: randomized controlled trials (RCTs). The exclusion criteria were as follows: (a) participants younger than 10 years old and older than 19 years old; (b) studies involving only a single exercise intervention; (c) articles not classified as RCTs; (d) studies with incomplete data; and (e) studies that did not include injury incidence rates.

### 2.3 Information sources and search strategy

A systematically search was conducted across five databases for this study; PubMed, Web of Science, Embase, EBSCOhost, and the Cochrane Library. The search covered the period from each database's inception to July 20 2024, without language restrictions. To ensure a comprehensive collection of relevant literature, reference lists from previous reviews were also screened and relevant studies were included. A detailed search strategy for each database is provided in Supplementary Table 1.

### 2.4 Extraction of data

The extracted data from the included articles consisted of the following: (1) basic information about the study (author's name, year of publication); (2) participant characteristics (age, sex, number); (3) duration of the training program; (4) compliance; (5) study design; (6) time of intervention; and (7) training program. Interventions were categorized into six types: (i) warm-up, (ii) stretching, (iii) plyometric/jumping exercises (grouped together), (iv) strength training, (v) balance training, and (vi) agility exercises. In addition, data were collected on outcome measures, including injury incidence rate to specific parts of the body (total injuries, lower-extremity injuries, knee injuries, ankle injuries, and upper-extremity injuries) and injury mechanisms (acute and overuse injuries). For articles with incomplete data, the corresponding authors were contacted for clarification. Data extraction was conducted collaboratively by two independent reviewers (HL and XL).

### 2.5 Research quality assessment

The Cochrane Risk of Bias Assessment Tool was employed to evaluate the risk of bias in the included studies (23). This assessment covered six domains: selection bias, performance bias, detection bias, attrition bias, reporting bias, and other bias. Each type of bias was judged according to its specific criteria, and the overall risk of bias for each study was categorized as low risk, unclear risk, or high risk. The quality of evidence was assessed using the Grading of Recommendations Assessment, Development, and Evaluation (GRADE) system (24). This evaluation considered five key domains: risk of bias, inconsistency, indirectness, imprecision, and publication bias. Each domain was rated based on established criteria to determine whether it was classified as serious or not serious. The criteria were as follows: (1) Risk of bias: assessed using the Cochrane Risk of Bias Assessment Tool; (2) Inconsistency: degree of heterogeneity between studies—high or low; (3) Indirectness: indirect comparison between studies; (4) Imprecision: fewer studies/participants included or consistency of results across studies; (5) Publication bias: Egger's test and funnel plot analysis used when ≥10 studies were included. Based on these evaluations, the overall quality of evidence was graded as high, moderate, low, or very low. Risk of Bias and GRADE assessments were conducted independently by two reviewers (HL and XL). In cases of disagreement, a third reviewer was consulted to resolve discrepancies through discussion. Inter-rater reliability was assessed using Cohen's Kappa. The Kappa values for Risk of Bias and Grade were 0.685 and 0.841, respectively, indicating substantial inter-rater agreement (25).

### 2.6 Data analysis and synthesis of results

The incidence rate ratio (IRR) with 95% confidence intervals (CIs) for injuries to different body regions (total injuries, lower-extremity injuries, knee injuries, ankle injuries, and upper-extremity injuries) and injury mechanisms (acute injuries and overuse injuries) was extracted and analyzed. Natural logarithm transformations were applied to all IRRs, and pooled estimates across studies were calculated using a random-effects inverse–variance meta-analysis (26). A random-effects model was selected over a fixed-effects model because the fixed-effects model assumes a common effect size to make the analysis reasonable, but there is usually no reason to assume that they are completely identical. In contrast, the random-effects model allows for the possibility of a distribution of true effects, making it more appropriate for this study (27).

Data were merged and analyzed using StataMP 17 software, and forest plots were generated. The inverse–variance random-effects model was used for statistical analysis of 95% CI and IRR (27). The *I*^2^ statistic was used to assess heterogeneity, with values of 25%, 50%, and 75% indicating low, moderate, and high heterogeneity, respectively. Cochran's *Q* test was also performed to evaluate heterogeneity among the studies, with *p* < 0.1 indicating high heterogeneity and *p* > 0.1 indicating low heterogeneity (28, 29). To explore potential influencing factors and identify the most effective training program, subgroup analyses were conducted. In the subgroup analysis of total injuries, sex was categorized into three subgroups (female, male, and combined female and male) and training duration was classified into two subgroups: ≥20 and <20 min. Six subgroups of training programs were defined (Supplementary Table2): (1) warm-up + stretching + jump/plyometrics + strength + balance + agility; (2) warm-up + jump/plyometrics + strength + balance + agility; (3) warm-up + jump/plyometrics + strength + balance; (4) jump/plyometrics + strength + balance; (5) warm-up + stretching + balance; and (6) strength + balance. Publication bias tests and sensitivity analyses were used to evaluate the stability and reliability of the results.

## 3 Results

### 3.1 Literature search results

**Figure 1:**
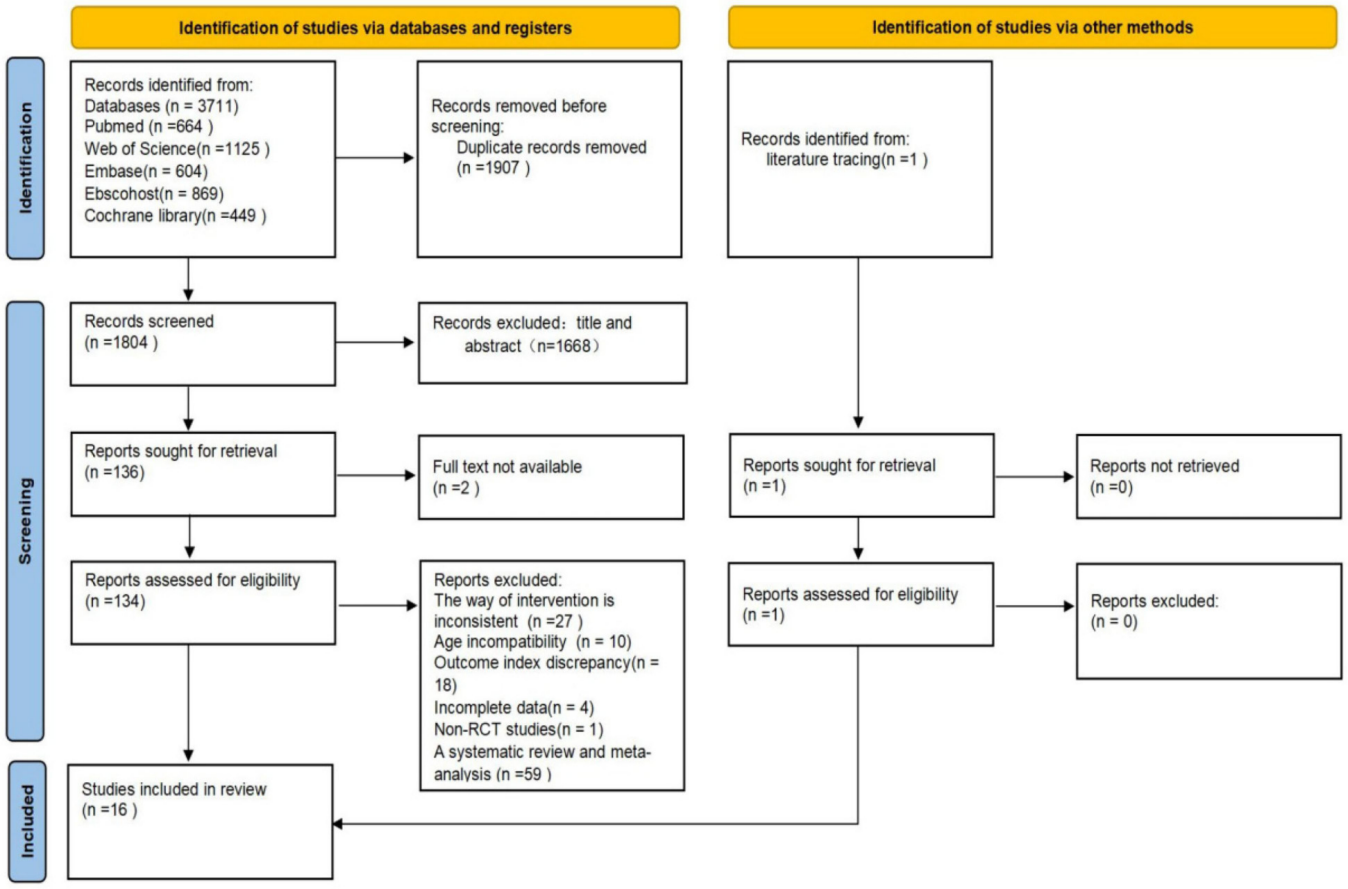
Flow chart of literature screening.

A total of 3,711 articles were retrieved from the database, of which 1,804 remained after duplicates were removed. Following a review of titles and abstracts, 136 articles were identified for further assessment. Two articles were excluded due to the inability to obtain the full text, and 15 articles were included after a full-text review based on predefined eligibility criteria. An additional article was identified through citation tracking, resulting in a total of 16 articles included in the meta-analysis. Reviewer 1 (HL) conducted an initial screening of the Embase, EBSCOhost, and Cochrane Library databases, identifying 1,922 studies. Reviewer 2 (XL) performed an initial screening of the PubMed and Web of Science databases, identifying 1,789 studies. Subsequently, both reviewers independently screened the studies according to the inclusion and exclusion criteria to avoid potential interference. For studies raising questions, the two reviewers discussed to reach consensus. If consensus could not be reached, a third reviewer (LY) served as an arbitrator, whose decision was final. The detailed screening process is illustrated in Figure 1.

### 3.2 The basic characteristics of included studies

All 16 included studies were randomized controlled trials, with one study designed as a three-arm trial (30). Among these studies, four included only male participants (31–34), six included only female participants (35–40), and six included both male and female participants (30, 41–45). The total sample size was 16,029 participants, with girls accounting for 79% and boys accounting for 21%. The intervention durations ranged from as short as 6 weeks (40) to as long as 1 year (30, 42, 45). The training session durations varied between 10 min (30, 35) and 60 min (40). Participant age ranges were reported in 15 studies, while one study provided only the mean age (39), and all participants were between 10 and 19 years old. The 16 articles all reported injuries to different parts of the body. In terms of injury mechanism, a total of 10 studies reported acute injury (31, 32, 36–39, 42–45) and 7 studies reported overuse injury (31, 32, 36, 37, 39, 43, 44). Compliance with the intervention program was reported in nine studies (31–33, 36, 37, 39, 42–44), while the remaining seven studies did not report compliance (30, 34, 35, 38, 40, 41, 45). The training programs for the intervention groups included stretching, jumping/plyometric exercises, strength training, balance training, and agility training. In contrast, the control groups performed usual warm-up exercises. The specific characteristics and details of the included studies are summarized in Table 1.

**Table 1:** Summary of the basic characteristics of the included studies.

Reference	Sex (age/years)	Training program	Number of players	Time of intervention, duration of training program	Compliance	Type of study	Outcome measures (exposure time/h)	Site/mechanism of injury (number of injuries/exposure time) IG CG
Achenbach et al. (41)	Female and male (13–18)	IG: Jump exercises, landing exercises, proprioceptive exercises, plyometric exercises and strength exercises	A: 279I	1 season (>10 weeks), 15 min	NA	A cluster-RCT	IG:2 6278	Total 50/26,278 32/17,929
			G: 168				CG:1 7,929	Lower extremity 28/26,278 22/17,929
			CG: 111					Knee 8/26,278 7/17,929
		CG: A usual warm-up training						Ankle 11/26,278 8/17,929
								Upper extremity 16/26,278 7/17,929
Belamjahad et al. (40)	Female (16–18)	IG: Warm-up, strength, plyometrics, change-of-direction speed, core stability, and balance	A: 24	6 weeks, 40–60 min	NA	RCT	IG: 4,485	Total 23/4,485 53/4,485
			IG: 12				CG: 4,485	Lower extremity 21/4,485 50/4,485
		CG: A traditional pre-season training program	CG: 12					
Emery and Meeuwisse (45)	Female and male (13–18)	IG: Warm-up (aerobic and stretching), neuromuscular training (strength, agility, balance, jump), and a home exercise program using a wobble board	A: 744	1 year, 30 min	NA	A cluster-RCT	IG: 24,051	Total 50/24,051 79/23,597
			IG: 380				CG: 23,597	Lower extremity 42/24,051 60/23,597
		CG: A standardized warm-up training						Knee 3/24,051 8/23,597
			CG: 364					Ankle 14/24,051 27/23,597
								Acute 42/24,051 72/23,597
LaBella et al.^a^ (39)	Female (IG: 16.19 ± 1.53; CG: 16.22 ± 1.06)	IG: Neuromuscular warm-up (jog, dynamic motion, strengthening exercises, plyometrics, agility runs)	A: 855	1 season, 20 min	80%	A cluster-RCT	IG: 20,345	Knee 6/20,345 11/12,467
			IG: 485				CG: 12,467	Ankle 7/20,345 11/12,467
								Acute 18/20,345 32/12,467
		CG: A usual warm-up training	CG: 370					Overuse 11/20,345 14/12,467
Obërtinca et al. (31)	Male (13–19)	IG: Warm-up, funball (balance, core stability, hamstring muscles eccentrics, gluteal muscle activation, plyometrics, running/sprinting, games) program	A: 1,027	9 months, 15–20 min	72.2%	A cluster-RCT	IG: 53,454	Total 132/53,454 187/52,938
							CG: 52,938	Lower extremity 108/53,454 159/52,938
			IG: 524					Knee 26/53,454 36/52,938
			CG: 503					Ankle 23/53,454 34/52,938
		CG: A usual warm-up training						Upper extremity 14/53,454 18/52,938
								Acute 114/53,454 165/52,938
								Overuse 18/53,454 22/52,938
Owoeye et al. (32)	Male (14–19)	IG: It consisted of three parts (I. slow running combined with active stretching; II. strength, plyometrics, balance; III. advanced running exercises)	A: 416	6 months, 20 min	60%	A cluster-RCT	IG: 51,017	Total 36/51,017 94/61,045
			IG: 212				CG: 61,045	Lower extremity 26/51,017 76/61,045
		CG: A usual non-structured warm-up						Knee 12/51,017 21/61,045
			CG: 204					Ankle 10/51,017 30/61,045
								Upper extremity 10/51,017 18/61,045
								Acute 34/51,017 80/61,045
								Overuse 2/51,017 14/61,045
Waldén et al. (38)	Female (12–17)	IG: The six exercises were a one legged knee squat, a pelvic lift, a two legged knee squat, the bench, the lunge, and jump/landing technique	A: 4,564	7 months, 15 min	NA	A cluster-RCT	IG: 149,214	Knee 49/149,214 47/129,084
							CG: 129,084	Acute 48/149,214 44/129,084
			IG: 2,479					
		CG: A usual training	CG: 2,085					
Steffen et al. (37)	Female (13–17)	IG: Jogging and a structured warm-up program (core stability, balance, plyometrics, strength)	A: 2,020	8 months, 20 min	24.1%	A cluster-RCT	IG: 66,423	Total 242/66,423 241/65,725
							CG: 65,725	Lower extremity 181/66,423 173/65,725
			IG: 1,073					
		CG: A usual warm-up training						Knee 37/66,423 30/65,725
			CG: 947					
								Ankle 79/66,423 74/65,725
								Acute 211/66,423 210/65,725
								Overuse 31/66,423 31/65,725
Asker et al.^b^ (30)	Female and male (14–19)	IG1: Shoulder control program (shoulder strength/control, upper body mobility, the diver with one arm in overhead positions, trunk rotational strength, handball throwing)	A: 627	1 year, 10–15 min	NA	A three-armed cluster-RCT	IG1: 22,650	Knee 77/22,650 108/23,600 93/25,500
							IG2: 25,500	
							CG: 23,600	
			IG1: 199					
			IG2: 216					
			CG: 212					
		IG2: Knee control program (one legged knee squats, pelvic lifts, two legged knee squats, the bench, lunges, jumping/landing)						
		CG: A usual training						
Emery et al. (42)	Female and male (IG: 13–18; CG: 12–18)	IG: Warm-up (aerobic and stretching), an additional sport-specific balance training and a home exercise program using a wobble board	A: 920	1 year, 35 min	60.3%	A cluster-RCT	IG: 39,369	Total 130/39,369 141/34,955
			IG: 494				CG: 34,955	Lower extremity 106/39,369 111/34,955
								Ankle 62/39,369 76/34,955
		CG: A standardized warm-up program	CG: 426					Acute 109/39,369 134/34,955
Åkerlund et al.^b^ (43)	Female and male (12–17)	IG: Warm-up, one legged knee squat, pelvic lift, two legged knee squat, the bench, the lunge, jump/landing	A: 471	26 weeks, 15–20 min	84%	A cluster-RCT	IG: 16,280	Total 197/16,280 152/8,128
			IG: 301				CG: 8,128	Lower extremity 155/16,280 126/8,128
								Knee 87/16,280 57/8,128
		CG: A usual training	CG: 170					Ankle 10/16,280 18/8,128
								Upper extremity 12/16,280 10/8,128
								Acute 52/16,280 47/8,128
								Overuse 145/16,280 105/8,128
Soligard et al. (36)	Female (13–17)	IG: It consisted of three parts (I. slow running combined with active stretching; II. strength, plyometrics, balance; III. advanced running exercises)	A: 1,892	8 months, 20 min	77%	A cluster-RCT	IG: 49,899	Total 161/49,899 215/45,428
								Lower extremity 124/49,899 158/45,428
			IG: 1,055				CG: 45,428	Knee 35/49,899 58/45,428
			CG: 837					Ankle 51/49,899 52/45,428
		CG: A usual training						Acute 136/49,899 163/45,428
								Overuse 25/49,899 52/45,428
Olsen et al. (44)	Female and male (15–17)	IG: It consisted of exercises with the ball, including the use of the wobble board and balance mat, for warm-up, technique (planting and cutting/jump shot landings), balance, strength	A: 1,837	8 months, 15–20 min	87%	A cluster-RCT	IG: 93,812	Total 103/93,812 195/87,483
							CG: 87,483	Acute 85/93,812 156/87,483
			IG: 958					
		CG: A usual training	CG: 879					Overuse 18/93,812 39/87,483
Zarei et al. (33)	Male (14–16)	IG: It consisted of three parts (I. slow running combined with active stretching; II. strength, plyometrics, balance; III. advanced running exercises)	A: 66	30 weeks, 20–25 min	74.5%	A cluster-RCT	IG: 4,078	Total 12/4,078 17/3,968
			IG: 34				CG: 3,968	
		CG: A regular warm-up training	CG: 32					
Wedderkopp et al. (35)	Female (16–18)	IG: The use of an ankle disk and 2 or more functional activities for all major muscle groups	A: 237	10 months, 10–15 min	NA	A cluster-RCT	IG: 14,578	Total 14/14,578 66/17,945
			IG: 111				CG: 17,945	Lower extremity 10/14,578 36/17,945
								Knee 2/14,578 8/17,945
			CG: 126					Ankle 6/14,578 23/17,945
		CG: A usual Training						Upper extremity 4/14,578 20/17,945
Farhan et al.^b^ (34)	Male (16–18)	IG: The exercises focus on core stabilization, eccentric training of thigh muscles, proprioceptive training, dynamic stabilization, and plyometrics	A: 50	12 weeks, 15–20 min	NA	RCT	IG: 9,375	Ankle 9/9,375 20/9,722
		CG: A regular training	IG: 25				CG: 9,722	
			CG: 25					

IG, intervention group; CG, control group; A, all; NA, not available.

a The study collected only basic information from 855 participants.

b The study did not provide the specific value for the exposure time, so the estimated value was obtained through calculation.

### 3.3 Quality assessment

**Figure 2:**
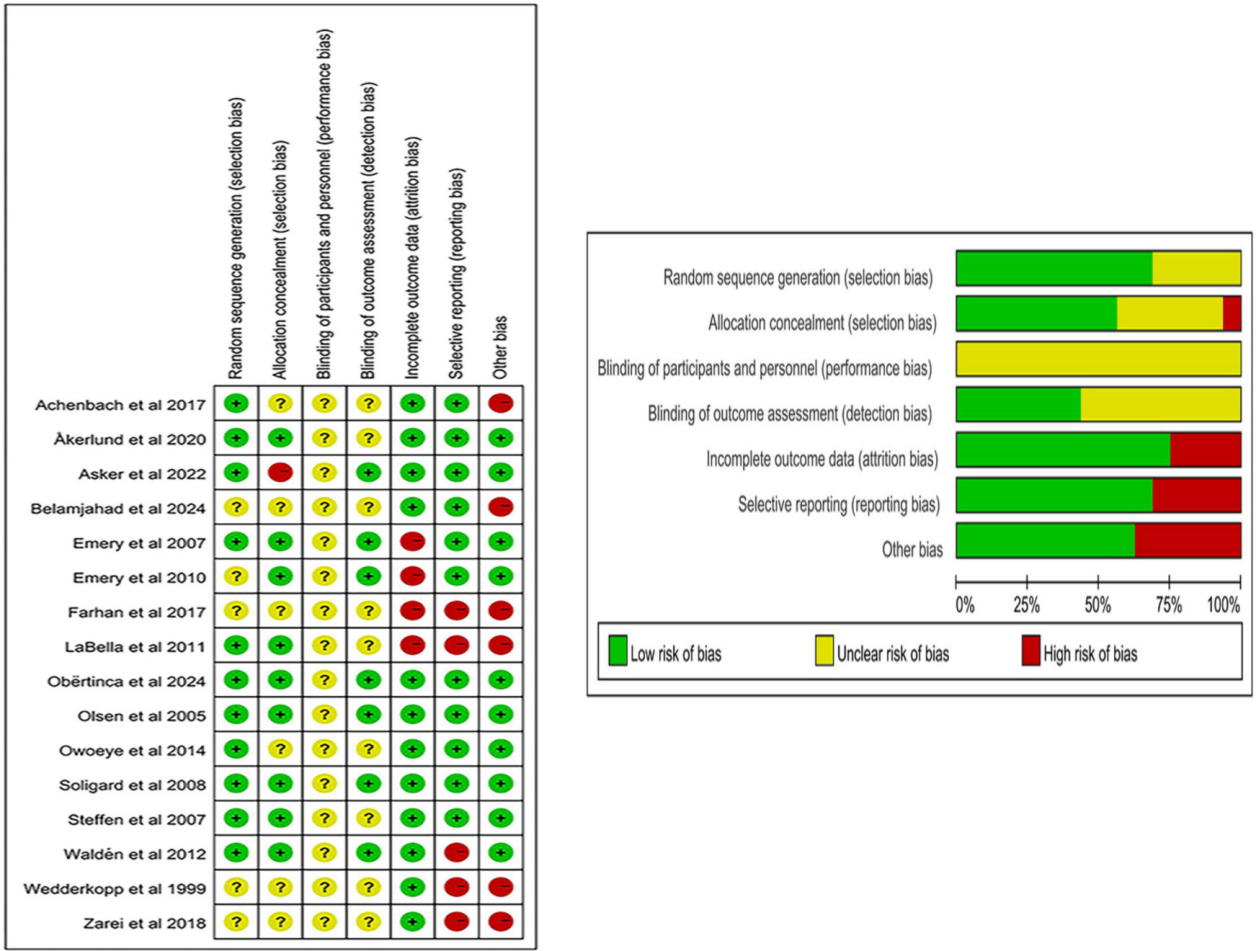
Risk of bias in the included literature.

In injury prevention intervention studies, blinding participants and researchers is almost not possible (46). Therefore, “Blinding of participants and personnel (performance bias)” was rated as an unclear risk (Figure 2). Given this premise, the potential impact of performance bias on study outcomes must be interpreted with caution. Among the 16 studies included, 11 studies described the generation of random sequences, while the remaining 5 studies did not give specific explanations.

### 3.4 Meta-analysis of the effects of multicomponent exercise injury prevention program

#### 3.4.1 Different parts of the body

**Figure 3:**
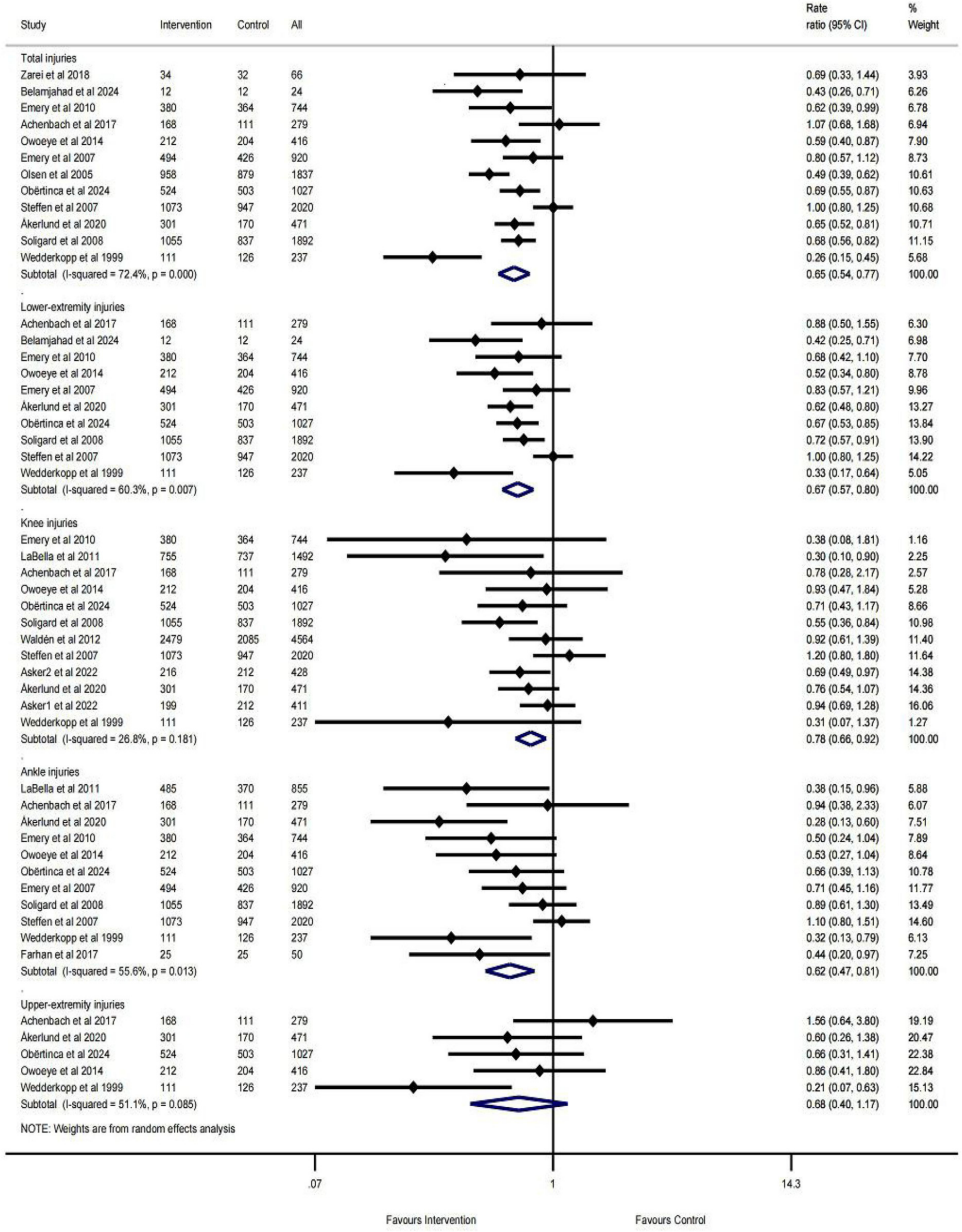
Pooled results for different parts of the body.

The pooled results (Figure 3) indicated that the multicomponent exercise injury prevention program had significant effects on total injuries (IRR = 0.65, 95% CI: 0.54–0.77, *I*^2^ = 72.4%, *p* = 0.000), lower-extremity injuries (IRR = 0.67, 95% CI: 0.57–0.80, *I*^2^ = 60.3%, *p* = 0.007), ankle injuries (IRR = 0.62, 95% CI: 0.47–0.81, *I*^2^ = 55.6%, *p* = 0.013), and upper-extremity injuries (IRR = 0.68, 95% CI: 0.40–1.17, *I*^2^ = 51.1%, *p* = 0.085), with the IRR consistently below 70%. In comparison, the prevention effect on knee injuries (IRR = 0.78, 95% CI: 0.66–0.92, *I*^2^ = 26.8%, *p* = 0.181) was more modest. Heterogeneity analysis revealed that the *I*^2^ values for total injuries, lower-extremity injuries, ankle injuries, and upper-extremity injuries were all above 50%.

#### 3.4.2 Injury mechanisms

**Figure 4:**
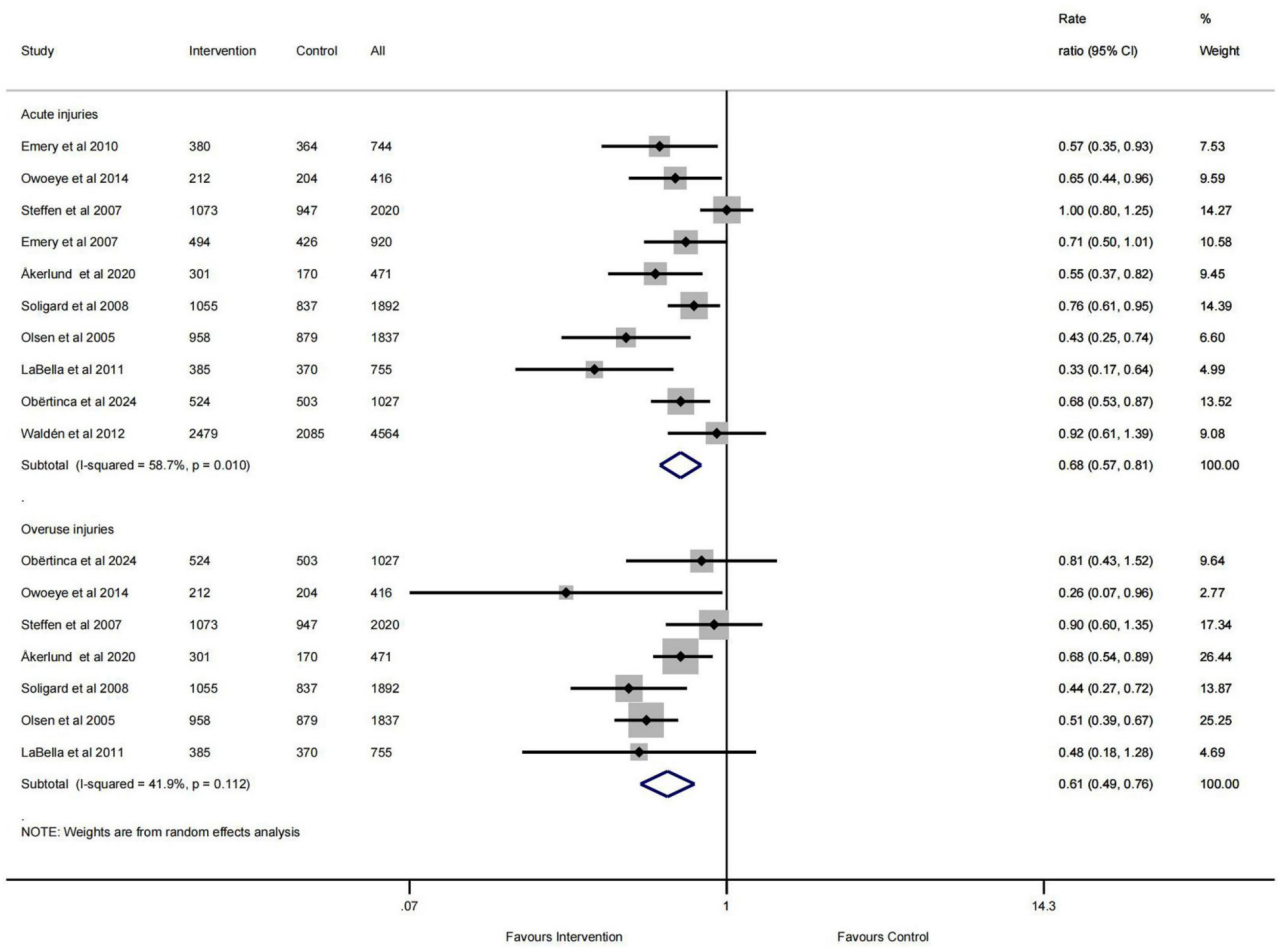
Pooled results for injury mechanisms.

The pooled results (Figure 4) demonstrated that multicomponent exercise injury prevention programs reduced acute injuries (IRR = 0.68, 95% CI: 0.57–0.81, *I*^2^ = 58.7%, *p* = 0.010) by 32% and overuse injuries (IRR = 0.61, 95% CI: 0.49–0.76, *I*^2^ = 41.9%, *p* = 0.112) by 39%.

#### 3.4.3 Subgroup analysis of total injuries

**Figure 5:**
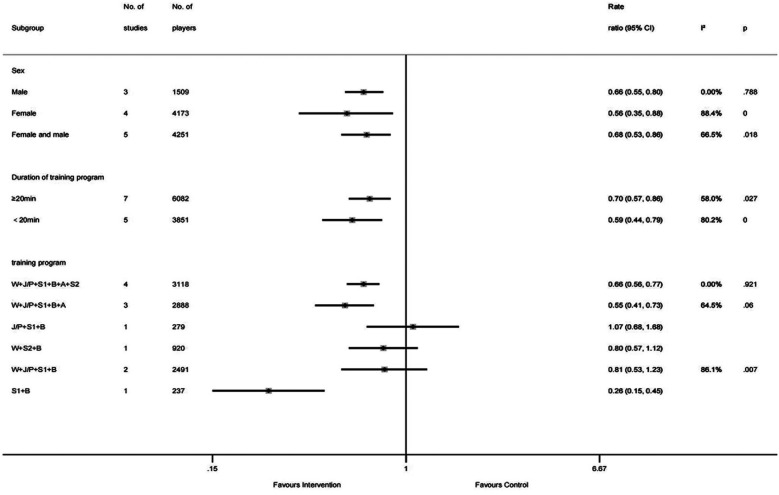
Pooled results for subgroup analysis. No., number; W, warm-up; J/P, jumping/plyometrics; S1, strength; B, balance; A, agility; S2, stretching.

Subgroup analysis of total injuries (Figure 5) revealed that girls showed greater benefits than boys and that interventions under 20 min were most effective. Specifically, the intervention was more effective in girls (IRR = 0.56, 95% CI: 0.35–0.88, *I*^2^ = 88.4%, *p* = 0.000) compared to boys (IRR = 0.66, 95% CI: 0.55–0.80, *I*^2^ = 0.00%, *p* = 0.788). In addition, interventions with a duration of under 20 min demonstrated a better effect (IRR = 0.59, 95% CI: 0.44–0.79, *I*^2^ = 80.2%, *p* = 0.000) than those lasting 20 min or more (IRR = 0.70, 95% CI: 0.57–0.86, *I*^2^ = 58.0%, *p* = 0.027). In studies that included two subgroups or more, the combination program of warm-up, jump/plyometric, strength, agility, stretching, and balance training (IRR = 0.55, 95% CI: 0.41–0.73, *I*^2^ = 64.5%, *p* = 0.06) showed the most favorable results, followed by the combination program of warm-up, jumping/plyometric, strength, agility, stretching, and balance training (IRR = 0.66, 95% CI: 0.56–0.77, *I*^2^ = 0.00%, *p* = 0.921). The combination program of warm-up, jumping/plyometric, strength, and balance training yielded the least effective result (IRR = 0.81, 95% CI: 0.53–1.23, *I*^2^ = 86.1%, *p* = 0.007). Moreover, the combination programs of strength and balance training (IRR = 0.26, 95% CI: 0.15–0.45); jumping/plyometric, strength, and balance training (IRR = 1.07, 95% CI: 0.68–1.68); and warm-up, stretching, and balance training (IRR = 0.80, 95% CI: 0.57–1.12) were each included in a single study. Subgroup analysis demonstrated that sex, training duration, and training program could not explain the high heterogeneity of total injury rates (*I*^2^ > 50% in all subgroups). The high heterogeneity of total injury rates may be due to differences in research methods or differences in the skill levels of athletes.

#### 3.4.4 Sensitivity analysis

Sensitivity analyses revealed stable heterogeneity for total injuries upon sequential study exclusion. However, exclusion of Steffen et al. significantly reduced heterogeneity (*I*^2^ < 50%) for lower-extremity, ankle, and acute injuries. This stems from low intervention adherence in their cohort, which attenuated preventive efficacy. For upper-extremity injuries, Wedderkopp et al. and Achenbach et al. were the primary heterogeneity sources, possibly due to the higher randomness of upper-extremity injury occurrence.

#### 3.4.5 Publication bias

The results of Egger's test for total injuries (*p* = 0.616), lower-extremity injuries (*p* = 0.194), and knee injuries (*p* = 0.242) indicated no publication bias, and the funnel plot showed symmetry. However, the funnel plots for ankle injuries and acute injuries were asymmetric, and Egger's test detected publication bias (*p* < 0.05) (47, 48) (refer to Supplementary Figure1). Subsequent analysis using the trim-and-fill method revealed that this publication bias did not influence the estimated results, as no trimming was performed and the data remained unchanged (29).

#### 3.4.6 Results of evidence grade evaluation

In the assessment of evidence quality for different injury types, we rated both total injuries and lower-extremity injuries as low due to serious issues with risk of bias and inconsistency. The rating for knee injuries was moderate, primarily due to serious concerns regarding risk of bias. The ratings for ankle injuries and acute injuries were very low, reflecting serious issues with risk of bias, inconsistency, and publication bias. The rating for upper-extremity injuries was also very low, with serious problems related to risk of bias, inconsistency, and imprecision. The rating for overuse injuries was moderate, with serious concerns regarding risk of bias. Detailed information can be found in Table 2.

**Table 2:** Evidence grade quality evaluation form.

Outcome measures	Number of studies (type)	Certainty assessment of evidence					RR (95% CI)	Evidence grade
		Risk of bias	Inconsistency	Indirectness	Imprecision	Publication bias		
Total injuries	12 (RCTs)	Serious	Serious	Not serious	Not serious	Not serious	0.65 (0.54–0.77)	⊕⊕○○ Low
Lower-extremity injuries	10 (RCTs)	Serious	Serious	Not serious	Not serious	Not serious	0.67 (0.57–0.80)	⊕⊕○○ Low
Knee injuries	12 (RCTs)	Serious	Not serious	Not serious	Not serious	Not serious	0.78 (0.66–0.92)	⊕⊕⊕○ Moderate
Ankle injuries	11 (RCTs)	Serious	Serious	Not serious	Not serious	Serious	0.62 (0.47–0.81)	⊕○○○ Very low
Upper-extremity injuries	5 (RCTs)	Serious	Serious	Not serious	Serious	NA	0.68 (0.40–1.17)	⊕○○○ Very low
Acute injuries	10 (RCTs)	Serious	Serious	Not serious	Not serious	Serious	0.68 (0.57–0.81)	⊕○○○ Very low
Overuse injuries	7 (RCTs)	Serious	Not serious	Not serious	Not serious	NA	0.61 (0.49–0.76)	⊕⊕⊕○ Moderate

According to the quality of the evidence from GRADE—High: we are confident that the true effect size is close to the estimated value; Moderate: we have moderate confidence in the estimated effect size; Low: we have limited confidence in the estimated effect size; Very low: we have little confidence in the estimated effect size.

## 4 Discussion

### 4.1 Research findings

This systematic review and meta-analysis evaluated the effectiveness of multicomponent exercise interventions in preventing injuries, including acute and overuse injuries, in adolescent team athletes. Through detailed subgroup analyses, we further elucidated the impact of variables such as sex, training duration, and the content of training programs on intervention outcomes. Importantly, this study identified the most effective combination of training programs for injury prevention.

### 4.2 Compared with previous studies

Previous studies have established that multicomponent exercise-based injury prevention programs are particularly effective in reducing total injuries among children (49). The present paper strictly defines the age of adolescents (10–19 years old) to prevent the influence of age bias on the study. Adolescence encompasses the rapid growth phase of puberty, during which there are significant differences in skeletal maturity, hormone levels, and neuromuscular control. Restricting the range reduces confounding bias caused by differences in developmental stages (50, 51). In contrast to prior reviews that included non-randomized controlled trials when evaluating the effectiveness of exercise-based injury prevention programs for adolescents (21), our study exclusively considered randomized controlled trials, which are considered the highest level of evidence (52). The pooled results of our study demonstrate an IRR of 0.65 for total injuries, consistent with the findings of recent studies (53, 54). When comparing our results to a review focused on exercise-based injury prevention in adolescents and children, several notable differences emerged. That review reported a rate ratio (RR) of 0.57 for lower-extremity injuries, 0.51 for ankle injuries, and 0.32 for knee injuries (20). In contrast, our study revealed an IRR of 0.67 for lower-extremity injuries, 0.62 for ankle injuries, and a significantly higher IRR of 0.78 for knee injuries. While the IRRs for lower-extremity and ankle injuries were approximately 10 percentage points higher than the RRs reported in the review, the IRR for knee injuries was notably 46 percentage points higher. This discrepancy may stem from the review's focus primarily on anterior cruciate ligament (ACL) injuries, whereas our study encompassed all types of knee injuries. A recent study examining the effectiveness of exercise-based injury prevention programs on reducing upper-extremity injuries in adolescents reported an IRR of 0.47 (53). On the contrary, the IRR for upper-extremity injuries in our study was 0.68, showing a significant difference of 21 percentage points. This difference may be attributed to the relatively small number of studies on upper-extremity injuries in our analysis and the poor injury effect of one of the studies included. Brunner et al. (19) investigated the effectiveness of multicomponent lower-extremity injuries prevention programs for team athletes and categorized the exercise elements into eight categories. In our study, exercise elements are reduced to six categories, among which jumping and plyometrics are grouped into one category. This simplification of the categories aimed to improve clarity while maintaining the differentiation of various exercise elements. Compared to previous studies, results were similar for girls (IRR 0.56 vs. 0.58) and different for boys (IRR 0.66 vs.0.56) (21). Differences between studies may stem from the fact that this study strictly limited the age range and types of sport included in the analysis. Our 20-min intervention threshold indicates that shorter intervention programs (<20 min) yield better results. This finding aligns with emerging evidence that interventions of ≤15 min can optimize injury prevention outcomes in adolescents, suggesting that appropriate intervention duration is more beneficial for preventing adolescent sports injuries (53). Multicomponent exercise intervention programs contain a variety of exercise elements and generally involve longer training durations. Therefore, this study set the intervention duration threshold at 20 min. A recurring key factor in sports injury prevention literature is the central role of intervention compliance. High-quality evidence across diverse populations and sports disciplines indicates that even well-designed, multicomponent exercise programs are significantly influenced by participant compliance (15). This underscores the critical importance of compliance data in evaluating intervention outcomes. The absence of compliance data in this study will consequently limit the validity of its assessment findings.

### 4.3 Total injuries, lower-extremity injuries, knee injuries, ankle injuries, and upper-extremity injuries

Following the intervention, injuries to different parts of the body were reduced to varying extents. Total injuries decreased by 35%, lower-extremity injuries by 33%, ankle injuries by 38%, and upper-extremity injuries by 32%. However, knee injuries were only reduced by 22%. The relatively lower prevention effect for knee injuries may be attributed to the intense physical demands of team sports, where athletes frequently face aggressive confrontations, change direction rapidly, and engage in sudden starts and stops, as well as jumping and landing. These movements often alter the knee's angle, increasing joint pressure during sagittal plane motion, which can lead to injury (55, 56). Furthermore, during planting and cutting maneuvers, valgus load increases, resulting in medial collateral ligament tension and lateral compression. This compressive load, combined with the anteriorly directed force vector generated by quadriceps contraction, results in displacement of the femur relative to the tibia. Specifically, the lateral femoral condyle moves posteriorly while the tibia translates anteriorly and undergoes internal rotation, significantly increasing ACL injury risk (57). Both the tibiofemoral and patellofemoral joints experience substantial compressive forces during normal activities. This occurs primarily because the line of action of relevant muscles lies close to the tibiofemoral joint's flexion-extension axis. This mechanical disadvantage necessitates greater muscle tension to counteract externally applied loads. In adolescents, inadequate muscle strength further elevates the risk of knee joint injury (58).

### 4.4 Acute injuries and overuse injuries

A 2021 study comparing the epidemiology of acute and overuse injuries in high school athletes found that, out of an estimated 17,434,646 injuries, 92.0% were acute and 8.0% were overuse injuries (59). These findings underscore the importance of focusing on acute injury prevention in adolescents. This study is the first to examine the effects of multicomponent exercise programs on both acute and overuse injuries in adolescent team athletes. Following intervention, acute injuries were reduced by 32%, while overuse injuries decreased by 39%.

### 4.5 Subgroup analysis of sex

Through subgroup analysis, we found that multicomponent exercise intervention programs were more effective in reducing total injuries among girls (IRR: 0.56) compared to boys (IRR: 0.66). This may be attributable to girls not exhibiting the same level of neuromuscular control as boys (60). Because multicomponent exercise programs are primarily designed to enhance neuromuscular control, they may be particularly beneficial for girls by strengthening their neuromuscular capabilities, which in turn reduces the likelihood of injury. In addition, this difference may be attributed to fundamental biomechanical differences between the sexes in dynamic movement patterns. Girls often demonstrate higher risk when performing movement-specific tasks, specifically manifesting as increased dynamic knee valgus angle, weakness or poor neuromuscular control in hip external rotators, and reduced trunk and hip flexion during landing (61, 62). Multicomponent intervention programs—including corrective jumping landing techniques, hamstring and core strength training, and trunk proprioception training—directly address these sex-specific biomechanical deficits. By optimizing the eccentric co-activation of the quadriceps and hamstrings during deceleration and enhancing coronal plane pelvic control, girls can achieve relatively greater improvements in joint stability, thereby explaining the reduced risk effect.

### 4.6 Subgroup analysis of training program duration

The results of the subgroup analysis revealed an interesting finding: Training programs lasting less than 20 min (IRR: 0.59) were more effective in preventing total injuries than those lasting 20 min or longer (IRR: 0.70). Several studies have compared the effects of warm-up exercises lasting approximately 15 min vs. those lasting 20 min or more. The findings suggest that 15-min warm-up routines lead to lower muscle fatigue, reduced blood lactate concentration, and improved exercise performance (63, 64). This may be the reason why training programs lasting less than 20 min are better for injury prevention.

### 4.7 Subgroup analysis of interventions

In a subgroup analysis of interventions, we found that balance training was included in all the training programs examined, while strength training was excluded in only one study (42). Notably, strength training primarily focused on lower-extremity strength, with particular emphasis on hamstring muscles and core strength. Studies have demonstrated that strengthening the hamstring muscles plays a critical role in preventing exercise-related injuries (65, 66). Therefore, it is recommended to incorporate both hamstring-focused and core-strengthening exercises alongside balance training in multicomponent exercise programs for injury prevention. Furthermore, when examining subgroups with two or more studies, the training combination (IRR: 0.55) of warm-up + jumping/plyometrics + strength + agility + balance demonstrated the most significant effect on total injury prevention. Previous studies have demonstrated the effectiveness of jumping/plyometric exercise, strength training, and balance training in preventing injuries (20, 67). In contrast, a single study did a combination of strength and balance training (IRR: 0.26), yielding the best results for total injury prevention. However, this finding was based on limited supporting research, and the study demonstrated poor-quality design. In addition, the training combination (IRR: 0.66) of warm-up + jumping/plyometric + strength + agility + balance + stretching also demonstrated a positive effect.

### 4.8 Strengths and limitations

This study focused on adolescents aged 10–19 years, deliberately excluding children under the age of 10. This approach was designed to more accurately evaluate the effects of intervention within this specific age group, thereby minimizing the potential influence of age-related variability on the results. Moreover, the study conducted subgroup analyses of different training combinations and training program durations, providing more detailed and practical guidance for adolescent team athletes. Furthermore, this study assessed the impact of multicomponent exercise intervention programs on both acute and overuse injuries among adolescent athletes participating in team sports.

Despite its strengths, this study has several unavoidable limitations that warrant attention. First, the quality of some studies included in the meta-analysis was suboptimal, posing potential risks to the accuracy and credibility of the overall findings. Second, the evidence grade for many outcome measures was generally low, indicating insufficient support for these measures and weakening the persuasiveness of the conclusions. Third, high heterogeneity was observed across the pooled results, likely attributable to divergent intervention adherence rates, variations in trial design (differing ratios of balance/strength/jumping components), and sport-specific demands (distinct biomechanical characteristics of football, basketball, and handball). This heterogeneity limits the reliability of interpretation. Lastly, the limited number of studies available restricted the scope of subgroup analyses, constraining the reliability of the results.

### 4.9 Research recommendations for the future

Based on the evidence quality ratings in our study, it is evident that additional high-quality research is urgently needed to further validate the effectiveness of multicomponent exercise injury prevention programs in adolescent team athletes. Future studies could focus on improving research quality by implementing blinded methods, randomizing study participants, and enhancing compliance. Currently, the majority of studies focus on football players, with limited studies evaluating other team sports, creating constraints in our broader understanding. Future research should target other team sports, comparing different sports or the same sport to identify the most suitable injury prevention strategies for each. Future research should not only continue to explore the optimal combination of multicomponent exercise interventions but also investigate the injury prevention effectiveness of different intervention sequences. We can combine different exercise elements in various ways for intervention comparisons to identify the optimal intervention sequence, which will help us further understand the intervention mechanisms. A major limitation of this study is the lack of compliance data, which prevented us from conducting subgroup analyses, potentially limiting the depth of our findings. Compliance data are essential for evaluating the effectiveness of interventions (68). The lack of compliance data has several critical implications for evidence-based practice (EBP). First, it prevents us from conducting planned subgroup analyses based on adherence levels, which are essential for discerning dose–response relationships and understanding the true potential of interventions under optimal conditions. Second, it introduces substantial uncertainty into our pooled effect estimates. This does not necessarily imply that multicomponent programs are ineffective, but rather that their effects may be diluted in practice due to suboptimal adherence. Therefore, any evidence-based practice recommendations derived from our findings must be interpreted with caution. Future studies can improve compliance by incorporating coach support, real-time feedback, reward design, and simplified intervention methods. Moreover, due to the inherent challenges of exercise intervention studies, blinding participants is often impractical, raising the possibility of contamination, as control group participants may be influenced by the training content of the intervention group. Future studies should consider methodological strategies to mitigate this risk (69). Therefore, in addition to a detailed investigation of the completion of the intervention group, completion of the control group must also be monitored, as well as conducting follow-up studies. Such approaches will allow for more accurate assessment of the actual effects of multicomponent exercise interventions and reduce the impact of potential biases on study results.

## 5 Conclusion

Multicomponent exercise intervention programs effectively reduce injuries across various body regions, including both acute and overuse injuries, in adolescent team athletes. Subgroup analysis of total injuries revealed sex-related differences and suggested that a training program lasting less than or equal to 20 min, along with a combination program of warm-up, jumping/plyometric, strength, agility, and balance training, is most effective for preventing exercise-related injuries. Critically, we strongly recommend that coaches and trainers prioritize multicomponent training programs that include balance training and strength training targeting the hamstrings and core muscles. However, these conclusions are drawn from low-quality evidence, underscoring the need for further high-quality studies to validate these findings.

## Data Availability

The original contributions presented in the study are included in the article/Supplementary Material, further inquiries can be directed to the corresponding author.
