# Supplementary material for: Effects of multicomponent exercise injury prevention programs on adolescent team athletes (10–19 years old): a systematic review and meta-analysis
Source: Front Pediatr. 2026 Jan 7;13:1561993. doi: 10.3389/fped.2025.1561993 (PMC12819658; doi:10.3389/fped.2025.1561993)
Supplement: Supplementary file 1 [file Table1.docx]

**Supplementary Table 1:** Meta-analysis retrieval strategy

| Pubmed | Search | Quantity(N) |
| --- | --- | --- |
| #1 | adolescent[MeSH Terms] | N=2,258,840 |
| #2 | （adolescen*[title/abstract]） OR （youth*[title/abstract]） OR （teen*[title/abstract]） | N=481,064 |
| #3 | #1OR#2 | N=2,388,078 |
| #4 | ((((((((((Team sport*[Title/Abstract]) OR (Soccer[Title/Abstract])) OR (Football[Title/Abstract])) OR (Basketball[Title/Abstract])) OR (Rugby[Title/Abstract])) OR (Volleyball[Title/Abstract])) OR (Baseball[Title/Abstract])) OR (Hockey[Title/Abstract])) OR (Water Polo[Title/Abstract])) OR (Handball[Title/Abstract])) OR (Cricket[Title/Abstract]) | N=45,975 |
| #5 | (athlete*[Title/Abstract]) OR (player*[Title/Abstract]) | N=145,730 |
| #6 | #4and#5 | N=31,651 |
| #7 | #3and#6 | N=10,564 |
| #8 | (((((((((((Multi-component[Title/Abstract]) OR (Multicomponent[Title/Abstract])) OR (strength[Title/Abstract])) OR (power[Title/Abstract])) OR (force[Title/Abstract])) OR (speed[Title/Abstract])) OR (velocity[Title/Abstract])) OR (balance[Title/Abstract])) OR (agility[Title/Abstract])) OR (Coordination[Title/Abstract])) OR (aerobic[Title/Abstract])) OR (Flexibility[Title/Abstract]) | N=1,996,409 |
| #9 | ((neuromuscular training[Title/Abstract]) OR (FIFA 11+[Title/Abstract])) OR (11+[Title/Abstract]) | N=1,777,939 |
| #10 | #8or#9 | N=3,653,936 |
| #11 | (((((((((Lower extremity[Title/Abstract])) OR (Lower limbs[Title/Abstract])) OR (Knee[Title/Abstract])) OR (Ankle[Title/Abstract])) OR (non-contact injuries[Title/Abstract])) OR (overall injuries[Title/Abstract])) OR (hamstring[Title/Abstract])) OR (ACL[Title/Abstract])) OR (injur*[Title/Abstract]) | N=1,338,703 |
| #12 | randomized controlled trial [Publication Type] | N=618,281 |
| #13 | controlled clinical trial [Publication Type] | N=708,932 |
| #14 | randomized [Title/Abstract] | N=713,790 |
| #15 | randomly [[Title/Abstract] | N=438,166 |
| #16 | trial [Title/Abstract] | N=829,517 |
| #17 | groups [Title/Abstract] | N=2,736,622 |
| #18 | #12or#13or#14or#15or#16or#17 | N=3,930,419 |
| #19 | animals [MeSH Terms] NOT humans [MeSH Terms] | N=58,23,309 |
| #20 | #18not#19 | N=3,410,130 |
| #21 | #7and#10and#11and#20 | N=664 |
| Web of science | Search | Quantity(N) |
| #1 | (((TS=(adolescent)) OR AB=(adolescen*)) OR AB=(youth*)) OR AB=(teen*) | N=[3,126,664](https://webofscience.clarivate.cn/wos/alldb/summary/927bbfc6-2c75-4551-9b09-dd7b342faff4-fb661c5d/times-cited-descending/1) |
| #2 | ((((((((((AB=(football)) OR AB=(soccer)) OR AB=(Team sport*)) OR AB=(Basketball)) OR AB=(Rugby)) OR AB=(Volleyball)) OR AB=(Baseball)) OR AB=(Hockey)) OR AB=(Water Polo)) OR AB=(Handball)) OR AB=(Cricket) | N=102,622 |
| #3 | (AB=(athlete*)) OR AB=(player*) | N=271,716 |
| #4 | #2 AND #3 | N=58,963 |
| #5 | #1 AND #4 | N=14,411 |
| #6 | ((((((((((((((((AB=(Multi-component)) OR AB=(Multicomponent)) OR AB=(strength)) OR AB=(power)) OR AB=(force)) OR AB=(speed)) OR AB=(velocity)) OR AB=(balance)) OR AB=(agility)) OR AB=(Coordination)) OR AB=(aerobic)) OR AB=(Flexibility)) OR AB=(neuromuscular training)) OR AB=(FIFA 11+)) OR AB=(11+)) OR AB=(injur*)) | N=10,861,355 |
| #7 | (((((((AB=(Lower extremity)) OR AB=(Lower limbs)) OR AB=(Knee)) OR AB=(Ankle)) OR AB=(non-contact injuries)) OR AB=(overall injuries)) OR AB=(hamstring)) OR AB=(ACL) | N=522,273 |
| #8 | (((((SO=(randomized controlled trial)) OR SO=(controlled clinical trial)) OR AB=(randomized)) OR AB=(randomly)) OR AB=(trial)) OR AB=(groups) | N=9,437,047 |
| #9 | (TS=(animals)) NOT TS=(humans) | N=6,160,934 |
| #10 | #8 NOT #9 | N=8,521,192 |
| #11 | #5 AND #6 AND #7 AND #10 | N=1,125 |
| Embase | Search | Quantity(N) |
| #1 | 'adolescent'/exp | N=2,001,533 |
| #2 | youth*:ab,ti OR adolescen*:ab,ti OR teen*:ab,ti | N=598,805 |
| #3 | #1 OR #2 | N=2,165,959 |
| #4 | 'team sport*':ab,ti OR soccer:ab,ti OR football:ab,ti OR basketabll:ab,ti OR rugby:ab,ti OR volleyball:ab,ti OR baseball:ab,ti OR hockey:ab,ti OR 'water polo':ab,ti OR handball:ab,ti OR cricket:ab,ti | N=47,309 |
| #5 | athlete*:ab,ti OR player*:ab,ti | N=174,543 |
| #6 | #4 AND #5 | N=32,044 |
| #7 | #3 AND #6 | N=9,492 |
| #8 | 'multi-component':ab,ti OR multicomponent:ab,ti OR strength:ab,ti OR power:ab,ti OR force:ab,ti OR speed:ab,ti OR velocity:ab,ti OR balance:ab,ti OR agility:ab,ti OR coordination:ab,ti OR aerobic:ab,ti OR flexibility:ab,ti | N=2,311,812 |
| #9 | 'neuromuscular training':ab,ti OR 'fifa 11+':ab,ti OR 11+:ab,ti | N=2,799,676 |
| #10 | #8 OR #9 | N=4,932,082 |
| #11 | 'lower extremity':ab,ti OR 'lower limbs':ab,ti OR knee:ab,ti OR ankle:ab,ti OR 'non-contact injuries':ab,ti OR 'overall injuries':ab,ti OR hamstring:ab,ti OR acl:ab,ti OR injur*:ab,ti | N=1,721,411 |
| #12 | 'randomized controlled trial':it OR 'controlled clinical trial':it OR randomized:ab,ti OR randomly:ab,ti OR trial:ab,ti OR groups:ab,ti | N=5,280,271 |
| #13 | 'animals'/exp NOT 'humans'/exp | N=6,154,069 |
| #14 | #12 NOT #13 | N=4,558,305 |
| #15 | #7 AND #10 AND #11 AND #14 | N=604 |
| Ebscohot | Search | Quantity(N) |
| S1 | SU adolescent | N=2,389,087 |
| S2 | AB adolescen* OR AB youth* OR AB teen* | N=1,460,778 |
| S3 | S1 OR S2 | N=3,464,965 |
| S4 | AB team sport* OR AB soccer OR AB football OR AB basketabll OR AB rugby OR AB volleyball OR AB baseball OR AB hockey OR AB water polo OR AB handball OR AB cricket | N=1,609,333 |
| S5 | AB athlete* OR AB player* | N=1,397,788 |
| S6 | S4 AND S5 | N=358,118 |
| S7 | S3 AND S6 | N=17,427 |
| S8 | AB multi-component OR AB multicomponent OR AB strength OR AB power OR AB force OR AB speed OR AB velocity OR AB balance OR AB agility OR AB coordination OR AB aerobic OR AB flexibility | N=9,136,725 |
| S9 | AB neuromuscular training OR AB fifa 11+ OR AB 11+ | N=2,155,435 |
| S10 | S8 OR S9 | N=11,133,581 |
| S11 | AB lower extremity OR AB lower limbs OR AB knee OR AB ankle OR AB non-contact injuries OR AB overall injuries OR AB hamstring OR AB acl OR AB injur* | N=2,358,817 |
| S12 | TX randomized controlled trial | N=1,649,183 |
| S13 | TX controlled clinical trial | N=312,635 |
| S14 | AB randomized | N=1,062,746 |
| S15 | AB randomly | N=805,271 |
| S16 | AB groups | N=11,223,756 |
| S17 | AB trial | N=2,430,998 |
| S18 | S12 OR S13 OR S14 OR S15 OR S16 OR S17 | N=14,047,339 |
| S19 | SU animals NOT SU humans | N=5,859,861 |
| S20 | S18 NOT S19 | N=13,167,264 |
| S21 | S7 AND S10 AND S11 AND S20 | N=869 |
| Cochrane library | Search | Quantity(N) |
| #1 | MeSH descriptor: [Adolescent] explode all trees | N=137,486 |
| #2 | (adolescen* OR youth* OR teen*):ti,ab,kw | N=178,798 |
| #3 | #1 OR #2 | N=178,798 |
| #4 | (team sport* OR football OR soccer OR rugby OR basketball OR volleyball OR baseball OR hockey OR water polo OR handball OR cricket):ti,ab,kw | N=6,285 |
| #5 | (athlete* OR player*):ti,ab,kw | N=13,274 |
| #6 | #4 AND #5 | N=4,524 |
| #7 | #3 AND #6 | N=1,548 |
| #8 | (multi-component OR multicomponent OR strength OR power OR force OR speed OR velocity OR balance OR agility OR coordination OR aerobic OR flexibility):ti,ab,kw | N=251,790 |
| #9 | (neuromuscular training OR fifa 11 OR 11):ti,ab,kw | N=285,067 |
| #10 | #8 OR #9 | N=490,119 |
| #11 | (lower extremity OR lower limbs OR knee OR ankle OR non-contact injuries OR overall injuries OR hamstring OR acl OR injur*):ti,ab,kw | N=148,348 |
| #12 | (randomized-controlled trial):ti,ab,kw | N=735,111 |
| #13 | (controlled clinical trial):ti,ab,kw | N=661,365 |
| #14 | (randomized):ti,ab,kw | N=1,186,492 |
| #15 | (randomly):ti,ab,kw | N=333,471 |
| #16 | (groups):ti,ab,kw | N=631,112 |
| #17 | (trial):ti,ab,kw | N=1,095,735 |
| #18 | #12 OR #13 OR #14 OR #15 OR #16 OR #17 | N=1,545,411 |
| #19 | (animals):ti,ab,kw NOT (humans):ti,ab,kw | N=5,174 |
| #20 | #18 NOT #19 | N=1,540,613 |
| #21 | #7 AND #10 AND #11 AND #20 | N=449 |

**Supplementary Figure1** Publication Bias Funnel Plot: The x-axis represents the effect size, the y-axis represents the standard error, and each circle represents an individual study.


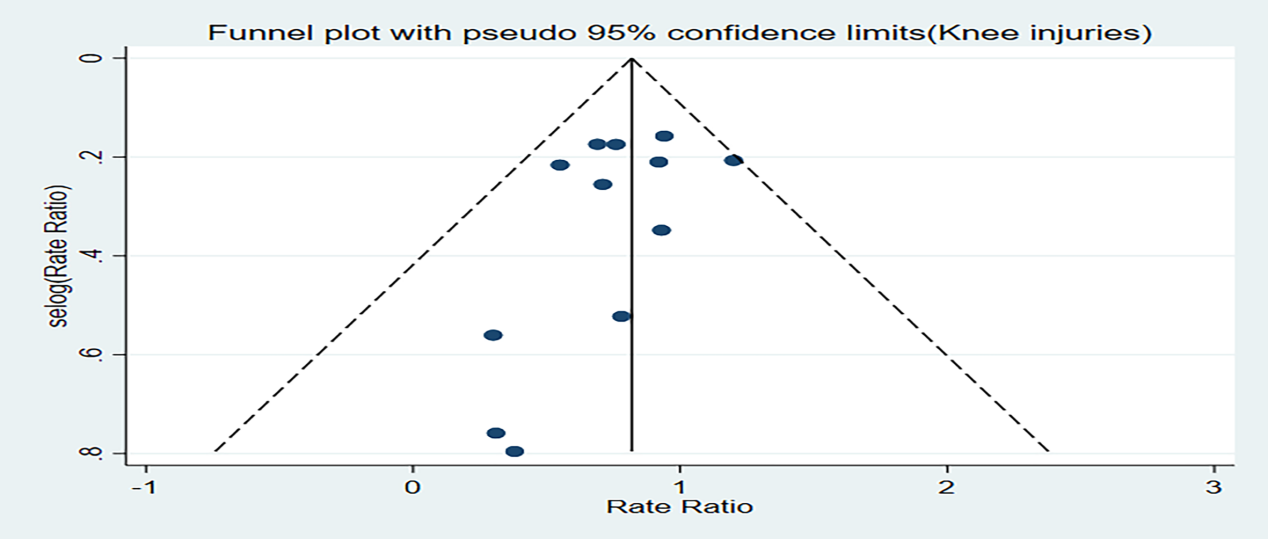

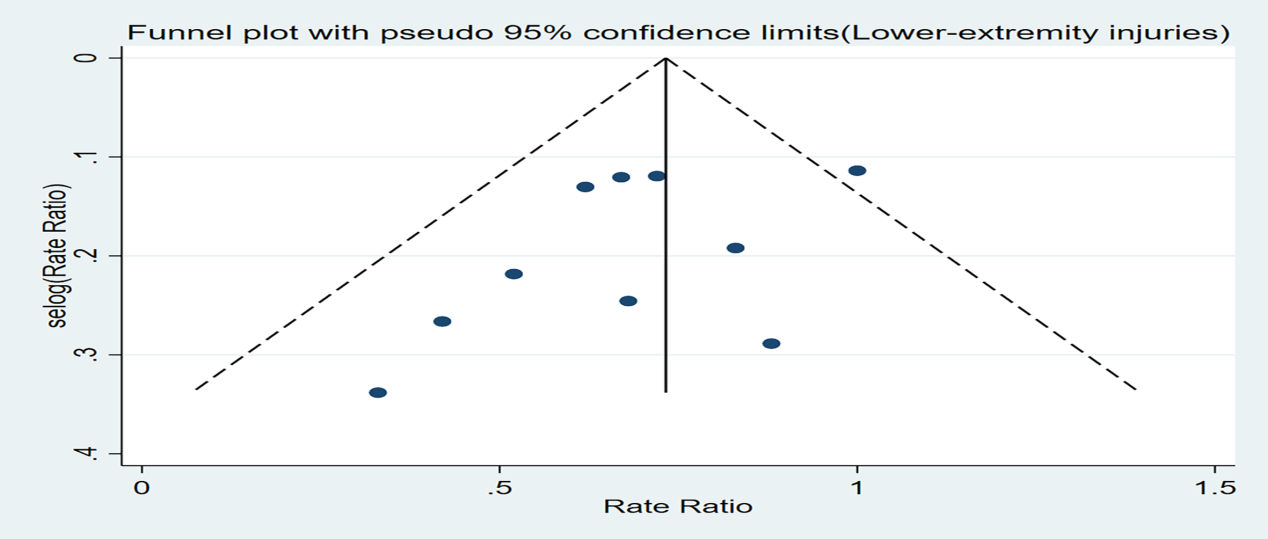

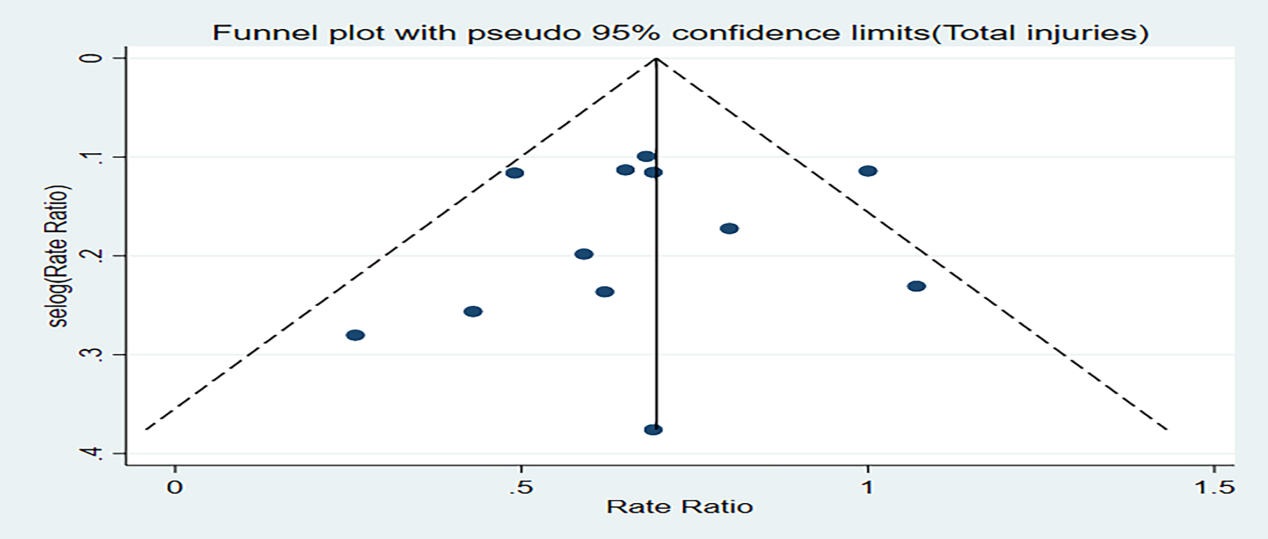


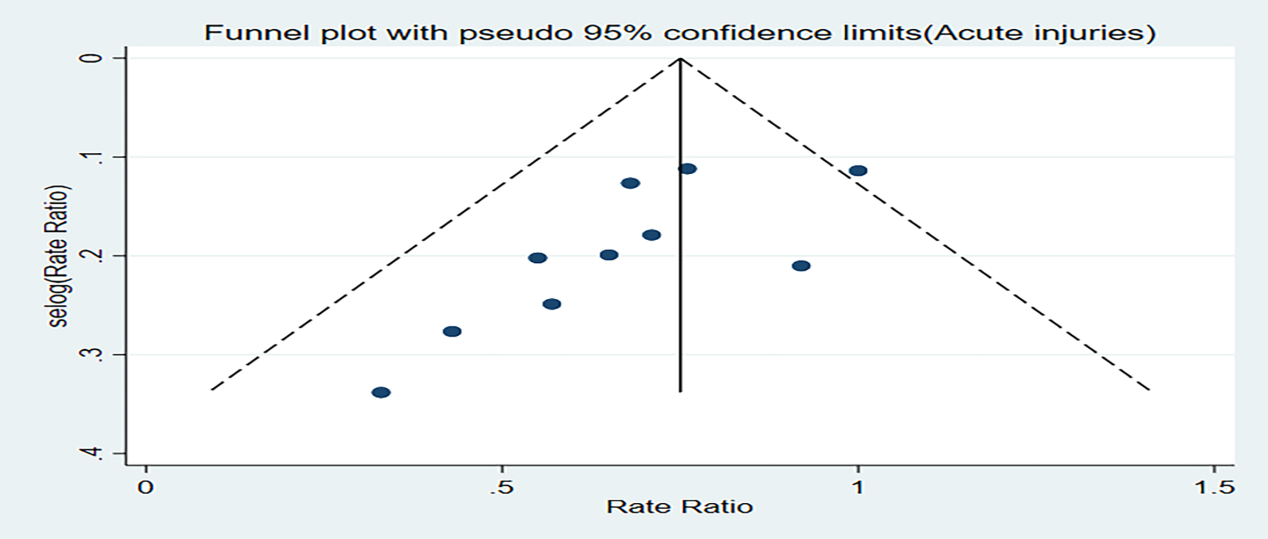

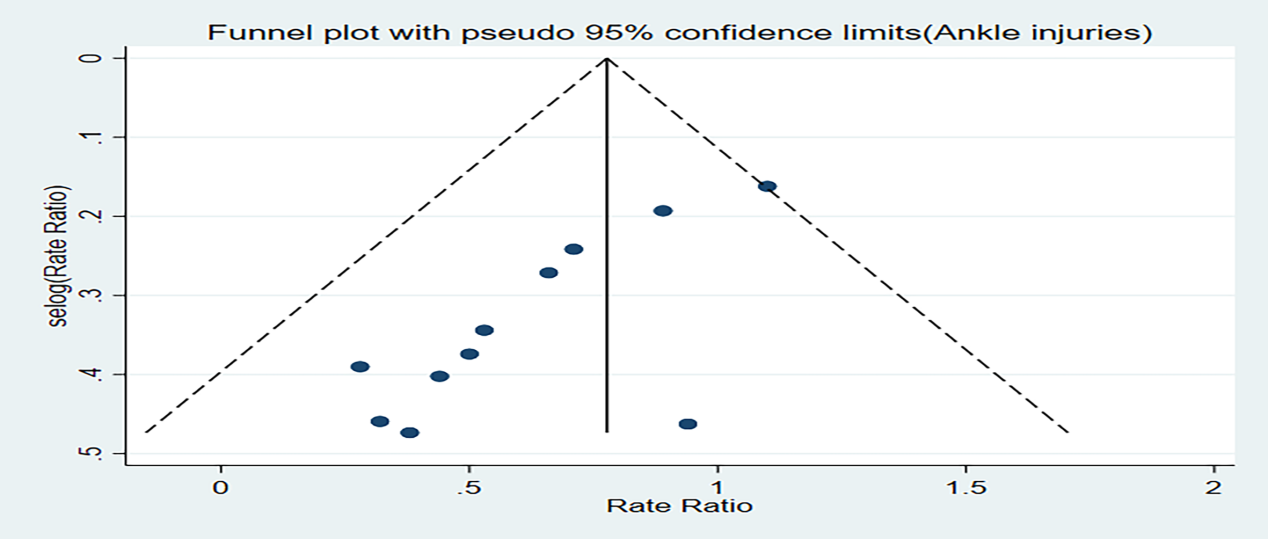


 **Supplementary Table2:** Combined training programmes for different studies

| Author/Year | warm-up | jump/plyometric | strength | agility | balance | stretching | quantities |
| --- | --- | --- | --- | --- | --- | --- | --- |
| Achenbach et al/2017 | × | √ | √ | × | √ | × | 3 |
| Belamjahad et al/2024 | √ | √ | √ | √ | √ | × | 5 |
| Emery et al/2010 | √ | √ | √ | √ | √ | √ | 6 |
| Obërtinca et al/2024 | √ | √ | √ | √ | √ | × | 5 |
| Owoeye et al/2014 | √ | √ | √ | √ | √ | √ | 6 |
| Steffen et al/2007 | √ | √ | √ | × | √ | × | 4 |
| Emery et al/2007 | √ | × | × | × | √ | √ | 3 |
| Åkerlund et al/2020 | √ | √ | √ | × | √ | × | 4 |
| Soligard et al/2008 | √ | √ | √ | √ | √ | √ | 6 |
| Olsen et al/2005 | √ | √ | √ | √ | √ | × | 5 |
| Zarei et al/2018 | √ | √ | √ | √ | √ | √ | 6 |
| Wedderkopp et al/1999 | × | × | √ | × | √ | × | 2 |

√: Represets the study that includes the training component; ×: Represents the study excluding the training component
