# Supplementary material for: Effects of multicomponent exercise injury prevention programs on adolescent team athletes (10–19 years old): a systematic review and meta-analysis
Source: Front Pediatr. 2026 Jan 7;13:1561993. doi: 10.3389/fped.2025.1561993 (PMC12819658; doi:10.3389/fped.2025.1561993)
Supplement: Supplementary file 2 [file Table2.docx]

**Table 1:** Meta-analysis retrieval strategy

| Pubmed | Search | Quantity(N) |
| --- | --- | --- |
| #1 | adolescent[MeSH Terms] | N=2,258,840 |
| #2 | （adolescen*[title/abstract]） OR （youth*[title/abstract]） OR （teen*[title/abstract]） | N=481,064 |
| #3 | #1OR#2 | N=2,388,078 |
| #4 | ((((((((((Team sport*[Title/Abstract]) OR (Soccer[Title/Abstract])) OR (Football[Title/Abstract])) OR (Basketball[Title/Abstract])) OR (Rugby[Title/Abstract])) OR (Volleyball[Title/Abstract])) OR (Baseball[Title/Abstract])) OR (Hockey[Title/Abstract])) OR (Water Polo[Title/Abstract])) OR (Handball[Title/Abstract])) OR (Cricket[Title/Abstract]) | N=45,975 |
| #5 | (athlete*[Title/Abstract]) OR (player*[Title/Abstract]) | N=145,730 |
| #6 | #4and#5 | N=31,651 |
| #7 | #3and#6 | N=10,564 |
| #8 | (((((((((((Multi-component[Title/Abstract]) OR (Multicomponent[Title/Abstract])) OR (strength[Title/Abstract])) OR (power[Title/Abstract])) OR (force[Title/Abstract])) OR (speed[Title/Abstract])) OR (velocity[Title/Abstract])) OR (balance[Title/Abstract])) OR (agility[Title/Abstract])) OR (Coordination[Title/Abstract])) OR (aerobic[Title/Abstract])) OR (Flexibility[Title/Abstract]) | N=1,996,409 |
| #9 | ((neuromuscular training[Title/Abstract]) OR (FIFA 11+[Title/Abstract])) OR (11+[Title/Abstract]) | N=1,777,939 |
| #10 | #8or#9 | N=3,653,936 |
| #11 | (((((((((Lower extremity[Title/Abstract])) OR (Lower limbs[Title/Abstract])) OR (Knee[Title/Abstract])) OR (Ankle[Title/Abstract])) OR (non-contact injuries[Title/Abstract])) OR (overall injuries[Title/Abstract])) OR (hamstring[Title/Abstract])) OR (ACL[Title/Abstract])) OR (injur*[Title/Abstract]) | N=1,338,703 |
| #12 | randomized controlled trial [Publication Type] | N=618,281 |
| #13 | controlled clinical trial [Publication Type] | N=708,932 |
| #14 | randomized [Title/Abstract] | N=713,790 |
| #15 | randomly [[Title/Abstract] | N=438,166 |
| #16 | trial [Title/Abstract] | N=829,517 |
| #17 | groups [Title/Abstract] | N=2,736,622 |
| #18 | #12or#13or#14or#15or#16or#17 | N=3,930,419 |
| #19 | animals [MeSH Terms] NOT humans [MeSH Terms] | N=58,23,309 |
| #20 | #18not#19 | N=3,410,130 |
| #21 | #7and#10and#11and#20 | N=664 |
| Web of science | Search | Quantity(N) |
| #1 | (((TS=(adolescent)) OR AB=(adolescen*)) OR AB=(youth*)) OR AB=(teen*) | N=[3,126,664](https://webofscience.clarivate.cn/wos/alldb/summary/927bbfc6-2c75-4551-9b09-dd7b342faff4-fb661c5d/times-cited-descending/1) |
| #2 | ((((((((((AB=(football)) OR AB=(soccer)) OR AB=(Team sport*)) OR AB=(Basketball)) OR AB=(Rugby)) OR AB=(Volleyball)) OR AB=(Baseball)) OR AB=(Hockey)) OR AB=(Water Polo)) OR AB=(Handball)) OR AB=(Cricket) | N=102,622 |
| #3 | (AB=(athlete*)) OR AB=(player*) | N=271,716 |
| #4 | #2 AND #3 | N=58,963 |
| #5 | #1 AND #4 | N=14,411 |
| #6 | ((((((((((((((((AB=(Multi-component)) OR AB=(Multicomponent)) OR AB=(strength)) OR AB=(power)) OR AB=(force)) OR AB=(speed)) OR AB=(velocity)) OR AB=(balance)) OR AB=(agility)) OR AB=(Coordination)) OR AB=(aerobic)) OR AB=(Flexibility)) OR AB=(neuromuscular training)) OR AB=(FIFA 11+)) OR AB=(11+)) OR AB=(injur*)) | N=10,861,355 |
| #7 | (((((((AB=(Lower extremity)) OR AB=(Lower limbs)) OR AB=(Knee)) OR AB=(Ankle)) OR AB=(non-contact injuries)) OR AB=(overall injuries)) OR AB=(hamstring)) OR AB=(ACL) | N=522,273 |
| #8 | (((((SO=(randomized controlled trial)) OR SO=(controlled clinical trial)) OR AB=(randomized)) OR AB=(randomly)) OR AB=(trial)) OR AB=(groups) | N=9,437,047 |
| #9 | (TS=(animals)) NOT TS=(humans) | N=6,160,934 |
| #10 | #8 NOT #9 | N=8,521,192 |
| #11 | #5 AND #6 AND #7 AND #10 | N=1,125 |
| Embase | Search | Quantity(N) |
| #1 | 'adolescent'/exp | N=2,001,533 |
| #2 | youth*:ab,ti OR adolescen*:ab,ti OR teen*:ab,ti | N=598,805 |
| #3 | #1 OR #2 | N=2,165,959 |
| #4 | 'team sport*':ab,ti OR soccer:ab,ti OR football:ab,ti OR basketabll:ab,ti OR rugby:ab,ti OR volleyball:ab,ti OR baseball:ab,ti OR hockey:ab,ti OR 'water polo':ab,ti OR handball:ab,ti OR cricket:ab,ti | N=47,309 |
| #5 | athlete*:ab,ti OR player*:ab,ti | N=174,543 |
| #6 | #4 AND #5 | N=32,044 |
| #7 | #3 AND #6 | N=9,492 |
| #8 | 'multi-component':ab,ti OR multicomponent:ab,ti OR strength:ab,ti OR power:ab,ti OR force:ab,ti OR speed:ab,ti OR velocity:ab,ti OR balance:ab,ti OR agility:ab,ti OR coordination:ab,ti OR aerobic:ab,ti OR flexibility:ab,ti | N=2,311,812 |
| #9 | 'neuromuscular training':ab,ti OR 'fifa 11+':ab,ti OR 11+:ab,ti | N=2,799,676 |
| #10 | #8 OR #9 | N=4,932,082 |
| #11 | 'lower extremity':ab,ti OR 'lower limbs':ab,ti OR knee:ab,ti OR ankle:ab,ti OR 'non-contact injuries':ab,ti OR 'overall injuries':ab,ti OR hamstring:ab,ti OR acl:ab,ti OR injur*:ab,ti | N=1,721,411 |
| #12 | 'randomized controlled trial':it OR 'controlled clinical trial':it OR randomized:ab,ti OR randomly:ab,ti OR trial:ab,ti OR groups:ab,ti | N=5,280,271 |
| #13 | 'animals'/exp NOT 'humans'/exp | N=6,154,069 |
| #14 | #12 NOT #13 | N=4,558,305 |
| #15 | #7 AND #10 AND #11 AND #14 | N=604 |
| Ebscohot | Search | Quantity(N) |
| S1 | SU adolescent | N=2,389,087 |
| S2 | AB adolescen* OR AB youth* OR AB teen* | N=1,460,778 |
| S3 | S1 OR S2 | N=3,464,965 |
| S4 | AB team sport* OR AB soccer OR AB football OR AB basketabll OR AB rugby OR AB volleyball OR AB baseball OR AB hockey OR AB water polo OR AB handball OR AB cricket | N=1,609,333 |
| S5 | AB athlete* OR AB player* | N=1,397,788 |
| S6 | S4 AND S5 | N=358,118 |
| S7 | S3 AND S6 | N=17,427 |
| S8 | AB multi-component OR AB multicomponent OR AB strength OR AB power OR AB force OR AB speed OR AB velocity OR AB balance OR AB agility OR AB coordination OR AB aerobic OR AB flexibility | N=9,136,725 |
| S9 | AB neuromuscular training OR AB fifa 11+ OR AB 11+ | N=2,155,435 |
| S10 | S8 OR S9 | N=11,133,581 |
| S11 | AB lower extremity OR AB lower limbs OR AB knee OR AB ankle OR AB non-contact injuries OR AB overall injuries OR AB hamstring OR AB acl OR AB injur* | N=2,358,817 |
| S12 | TX randomized controlled trial | N=1,649,183 |
| S13 | TX controlled clinical trial | N=312,635 |
| S14 | AB randomized | N=1,062,746 |
| S15 | AB randomly | N=805,271 |
| S16 | AB groups | N=11,223,756 |
| S17 | AB trial | N=2,430,998 |
| S18 | S12 OR S13 OR S14 OR S15 OR S16 OR S17 | N=14,047,339 |
| S19 | SU animals NOT SU humans | N=5,859,861 |
| S20 | S18 NOT S19 | N=13,167,264 |
| S21 | S7 AND S10 AND S11 AND S20 | N=869 |
| Cochrane library | Search | Quantity(N) |
| #1 | MeSH descriptor: [Adolescent] explode all trees | N=137,486 |
| #2 | (adolescen* OR youth* OR teen*):ti,ab,kw | N=178,798 |
| #3 | #1 OR #2 | N=178,798 |
| #4 | (team sport* OR football OR soccer OR rugby OR basketball OR volleyball OR baseball OR hockey OR water polo OR handball OR cricket):ti,ab,kw | N=6,285 |
| #5 | (athlete* OR player*):ti,ab,kw | N=13,274 |
| #6 | #4 AND #5 | N=4,524 |
| #7 | #3 AND #6 | N=1,548 |
| #8 | (multi-component OR multicomponent OR strength OR power OR force OR speed OR velocity OR balance OR agility OR coordination OR aerobic OR flexibility):ti,ab,kw | N=251,790 |
| #9 | (neuromuscular training OR fifa 11 OR 11):ti,ab,kw | N=285,067 |
| #10 | #8 OR #9 | N=490,119 |
| #11 | (lower extremity OR lower limbs OR knee OR ankle OR non-contact injuries OR overall injuries OR hamstring OR acl OR injur*):ti,ab,kw | N=148,348 |
| #12 | (randomized-controlled trial):ti,ab,kw | N=735,111 |
| #13 | (controlled clinical trial):ti,ab,kw | N=661,365 |
| #14 | (randomized):ti,ab,kw | N=1,186,492 |
| #15 | (randomly):ti,ab,kw | N=333,471 |
| #16 | (groups):ti,ab,kw | N=631,112 |
| #17 | (trial):ti,ab,kw | N=1,095,735 |
| #18 | #12 OR #13 OR #14 OR #15 OR #16 OR #17 | N=1,545,411 |
| #19 | (animals):ti,ab,kw NOT (humans):ti,ab,kw | N=5,174 |
| #20 | #18 NOT #19 | N=1,540,613 |
| #21 | #7 AND #10 AND #11 AND #20 | N=449 |

**Table2**：Summary of the basic characteristics of the included studies

| **Authour (Year)** | **Sex (Age/Years)** | **Training program** | **Number of Players** | **Time of intervention，Duration of training program** | **Compliance** | **Type of study** | **Outcome measures(Exposure time/h)** | **Site/mechanism of injury(Number of injuries/Exposure time)**    **IG CG** |
| --- | --- | --- | --- | --- | --- | --- | --- | --- |
| Achenbach et al (2017) | Female and male (13-18) | IG:Jump exercises, landing exercises, proprioceptive exercises,plyometric exercises and strength exercises CG:A usual warm-up training | A:279IG:168  CG:111 | 1 season (＞10weeks)，  15 min | NA | a cluster-RCT | IG:26278  CG:17929 | Total; 50/26278 32/17929  Low-extremity; 28/26278 22/17929  Knee; 8/26278 7/17929  Ankle; 11/26278 8/17929  Upper-extremity 16/26278 7/17929 |
| Belamjahad et al (2024) | Female (16-18) | IG:Warm-up,  strength, plyometrics, change-of-direction speed,core stability and balance   CG:A traditional pre-season training program | A:24 IG:12  CG:12 | 6 weeks， 40-60min | NA | RCT | IG:4485  CG:4485 | Total; 23/4485 53/4485  Low-extremity 21/4485 50/4485 |
| Emery et al (2010) | Female and male (13-18) | IG:Warm-up(aerobic and stretching), neuromuscular training (strength, agility, balance, jump) and a home exercise program using a wobble board CG:A standardised warm-up training | A:744 IG:380  CG:364 | 1 year， 30min | NA | a cluster-RCT | IG:24051  CG:23597 | Total; 50/24051 79/23597  Low-extremity; 42/24051 60/23597  Knee; 3/24051 8/23597  Ankle; 14/24051 27/23597  Acute 42/24051 72/23597 |
|  |  |  |  |  |  |  |  |  |
| LaBella et al^a^ (2011) | Female (IG:16.19±1.53 CG:16.22±1.06) | IG:Neuromuscular warm-up(jog, dynamic motion, strengthening exercises, plyometrics, agility runs) CG:A usual warm-up training | A:855 IG:485  CG:370 | 1 season， 20min | 80% | a cluster-RCT | IG:20345  CG:12467 | Knee; 6/20345 11/12467  Ankle; 7/20345 11/12467  Acute; 18/20345 32/12467  Overuse 11/20345 14/12467 |
| **Authour (Year)** | **Sex (Age/Years)** | **Training program** | **Number of Players** | **Time of intervention，Duration of training program** | **Compliance** | **Type of study** | **Outcome measures(Exposure time/h)** | **Site/mechanism of injury(Number of injuries/Exposure time)**    **IG CG** |
| Obertinca et al (2024) | Male (13-19) | IG:Warm-up,funball(balance，core stability, hamstring muscles eccentrics, gluteal muscle activation, plyometrics, running/sprinting, games)programme CG:A usual warm-up training | A:1027 IG:524  CG:503 | 9 months, 15-20min | 72.2% | a cluster-RCT | IG:53454  CG:52938 | Total; 132/53454 187/52938  Low-extremity;108/53454 159/52938  Knee; 26/53454 36/52938  Ankle; 23/53454 34/52938  Upper-extremity;14/53454 18/52938  Acute; 114/53454 165/52938  Overuse 18/53454 22/52938 |
| Owoeye et al (2014) | Male (14-19) | IG:It consisted of three parts (Ⅰ.slow running combined with active stretching Ⅱ.Strength,plyometrics,balance Ⅲ.advanced running exercises) CG:A usual non-structured warm-up | A:416 IG:212  CG:204 | 6 months, 20min | 60% | a cluster-RCT | IG:51017  CG:61045 | Total; 36/51017 94/61045  Low-extremity; 26/51017 76/61045  Knee; 12/51017 21/61045  Ankle; 10/51017 30/61045  Upper-extremity;10/51017 18/61045  Acute; 34/51017 80/61045  Overuse 2/51017 14/61045 |
| Waldén et al  (2012) | Female (12-17) | IG:The six exercises were a one legged knee squat, a pelvic lif  t, a two legged knee squat, the bench, the lunge, and jump/landing technique CG:A usual training | A:4564 IG:2479  CG:2085 | 7 months, 15min | NA | a  cluster-RCT | IG:149214  CG:129084 | Knee; 49/149214 47/129084  Acute 48/149214 44/129084 |
| Steffen et al (2007) | Female (13-17) | IG: Jogging and a structured  warm-up program(Core stability, Balance, Plyometrics, Strength) CG:A usual warm-up training | A:2020 IG:1073  CG:94  7 | 8 months,  20min | 24.1% | a cluster-RCT | IG:66423  CG:65725 | Total; 242/66423 241/65725  Low-extremity;181/66423 173/65725  Knee; 37/66423 30/65725  Ankle; 79/66423 74/65725  Acute; 211/66423 210/65725  Overuse 31/66423 31/65725 |
| **Authour (Year)** | **Sex (Age/Years)** | **Training program** | **Number of Players** | **Time of intervention，Duration of training program** | **Compliance** | **Type of study** | **Outcome measures(Exposure time/h)** | **Site/mechanism of injury(Number of injuries/Exposure time)**    **IG CG** |
| Asker et al^b^ (2022) | Female and male (14-19) | IG1:Shoulder control programme(shoulder strength/control,upper body mobility, the diver with one arm in overhead positions, trunk rotational strength, handball throwing) IG2:Knee control programme(one legged knee squats, pelvic lifts,two legged knee squats,the bench, lunges, jumping/landing) CG:A usual training | A:627 IG1:199 IG2:216  CG:212 | 1 year, 10-15min | NA | a three-armed cluster-RCT | IG1:22650 IG2:25500  CG:23600 | Knee 77/22650 108/23600  93/25500 |
| Emery et al (2007) | Female and male (IG:13-18 CG:12-18) | IG:Warm-up(aerobic and stretching), an additional sport-specific balance training and a home exercise program using a wobble board CG:A standardized warm-up program | A:920 IG:494  CG:426 | 1 year, 35min | 60.3% | a cluster-RCT | IG:39369  CG:34955 | Total; 130/39369 141/34955  Low-extremity;106/39369 111/34955  Ankle; 62/39369 76/34955  Acute 109/39369 134/34955 |
| Akerlund et al^b^ (2020) | Female and male (12-17) | IG: Warn-up, one legged knee squat, pelvic lift, two legged knee squat, the bench, the lunge, jump/landing CG:A usual training | A:471 IG:301  CG:170 | 26 weeks, 15-20min | 84% | a cluster-RCT | IG:16280  CG:8128 | Total; 197/16280 152/8128  Low-extremity; 155/16280 126/8128  Knee; 87/16280 57/8128  Ankle; 10/16280 18/8128  Upper-extremity;12/16280 10/8128  Acute; 52/16280 47/8128  Overuse 145/16280 105/8128 |
| **Authour (Year)** | **Sex (Age/Years)** | **Training program** | **Number of Players** | **Time of intervention，Duration of training program** | **Compliance** | **Type of study** | **Outcome measures(Exposure time/h)** | **Site/mechanism of injury(Number of injuries/Exposure time)**  **IG CG** |
| Soligard et al (2008) | Female (13-17) | IG:It consisted of three parts (Ⅰ.slow running combined with active stretching Ⅱ.strength,plyometrics,balance Ⅲ.advanced running exercises) CG:A usual training | A:1892 IG:1055  CG:837 | 8 months, 20min | 77% | a cluster-RCT | IG:49899  CG:45428 | Total; 161/49899 215/45428  Low-extremity;124/49899 158/45428  Knee; 35/49899 58/45428  Ankle; 51/49899 52/45428  Acute; 136/49899 163/45428  Overuse 25/49899 52/45428 |
| Olsen et al (2005) | Female and male (15-17) | IG:The consisted of exercises with the ball, including the use of the wobble board and balance mat, for warm-up, technique(Planting and cutting/Jump shot landings), balance, strength CG:A usual training | A:1837 IG:958  CG:879 | 8 months, 15-20min | 87% | a cluster-RCT | IG:93812  CG:87483 | Total; 103/93812 195/87483  Acute; 85/93812 156/87483  Overuse 18/93812 39/87483 |
| Zarei et al (2018) | Male (14-16) | IG:It consisted of three parts (Ⅰ.slow running combined with active stretching Ⅱ.Strength,plyometrics,balance Ⅲ.advanced running exercises) CG:A regular warm-up training | A:66 IG:34  CG:32 | 30 weeks, 20-25min | 74.5% | a cluster-RCT | IG:4078  CG:3968 | Total 12/4078 17/3968 |
| Wedderkopp et al (1999) | Female (16-18) | IG:The use of an ankle disk and 2 or more functional activities for all major muscle groups CG:A usual  Training | A:237 IG:111  CG:126 | 10 months, 10-15min | NA | a cluster-RCT | IG:14578  CG:17945 | Total; 14/14578 66/17945  Low-extremity; 10/14578 36/17945  Knee; 2/14578 8/17945  Ankle; 6/14578 23/17945  Upper-extremity 4/14578 20/17945 |
| **Authour (Year)** | **Sex (Age/Years)** | **Training program** | **Number of Players** | **Time of intervention，Duration of training program** | **Compliance** | **Type of study** | **Outcome measures(Exposure time/h)** | **Site/mechanism of injury(Number of injuries/Exposure time)**  **IG CG** |
| Farhan^b^ (2017) | Male (16-18) | IG:The exercises focus on core stabilisation, eccentric training of thigh muscles, proprioceptive training, dynamic stabilisation and plyometrics.  CG:A regular training | A:50 IG:25  CG:25 | 12 weeks, 15-20min | NA | RCT | IG:9375  CG:9722 | Ankle 9/9375 20/9722 |

IG: Intervention Group; CG: Control Group; A: All; NA: Not Available; a: The study collected only basic information from 855 participants; b: The study did not provide the specific value for the exposure time, so the estimated value was obtained through calculation.

**Table3:** Evidence grade quality evaluation form

| Outcome measures | Number of studies (Type) | Certainty assessment of evidence | | | | | RR(95%CI) | Evidence grade |
| --- | --- | --- | --- | --- | --- | --- | --- | --- |
|  |  | risk of bias | inconsistency | indirectness | imprecision | publication bias |  |  |
| Total injuries | 12 (RCT) | serious | serious | not serious | not serious | not serious | 0.65（0.54,0.77） | ⊕⊕○○ low |
| Lower-extremity injuries | 10 (RCT) | serious | serious | not serious | not serious | not serious | 0.67（0.57,0.80） | ⊕⊕○○ low |
| Knee injuries | 12 (RCT) | serious | not serious | not serious | not serious | not serious | 0.78（0.66,0.92） | ⊕⊕⊕○ moderate |
| Ankle injuries | 11 (RCT) | serious | serious | not serious | not serious | serious | 0.62（0.47,0.81） | ⊕○○○ very low |
| Upper-extremity injuries | 5 (RCT) | serious | serious | not serious | serious | NA | 0.68（0.40,1.17） | ⊕○○○ very low |
| Acute injuries | 9 (RCT) | serious | serious | not serious | not serious | serious | 0.68（0.57,0.81） | ⊕○○○ very low |
| Overuse injuries | 7 (RCT) | serious | not serious | not serious | not serious | NA | 0.61（0.49,0.76） | ⊕⊕⊕○ moderate |

According to the quality of the evidence from GRADE:
High: We are confident that the true effect size is close to the estimated value.
Moderate: We have moderate confidence in the estimated effect size.
Low: We have limited confidence in the estimated effect size.
Very low: We have little confidence in the estimated effect size.

 **Table4**: Combined training programmes for different studies

| Author/Year | warm-up | jump/plyometric | strength | agility | balance | stretching | quantities |
| --- | --- | --- | --- | --- | --- | --- | --- |
| Achenbach et al/2017 | × | √ | √ | × | √ | × | 3 |
| Belamjahad et al/2024 | √ | √ | √ | √ | √ | × | 5 |
| Emery et al/2010 | √ | √ | √ | √ | √ | √ | 6 |
| Obërtinca et al/2024 | √ | √ | √ | √ | √ | × | 5 |
| Owoeye et al/2014 | √ | √ | √ | √ | √ | √ | 6 |
| Steffen et al/2007 | √ | √ | √ | × | √ | × | 4 |
| Emery et al/2007 | √ | × | × | × | √ | √ | 3 |
| Åkerlund et al/2020 | √ | √ | √ | × | √ | × | 4 |
| Soligard et al/2008 | √ | √ | √ | √ | √ | √ | 6 |
| Olsen et al/2005 | √ | √ | √ | √ | √ | × | 5 |
| Zarei et al/2018 | √ | √ | √ | √ | √ | √ | 6 |
| Wedderkopp et al/1999 | × | × | √ | × | √ | × | 2 |

√: Represents the study that includes the training component; ×: Represents the study excluding the training component
